# Grain Inorganic Arsenic Content in Rice Managed Through Targeted Introgressions and Irrigation Management

**DOI:** 10.3389/fpls.2020.612054

**Published:** 2021-01-25

**Authors:** Cristina P. Fernández-Baca, Anna M. McClung, Jeremy D. Edwards, Eton E. Codling, Vangimalla R. Reddy, Jinyoung Y. Barnaby

**Affiliations:** ^1^United States Department of Agriculture, Agricultural Research Service, Dale Bumpers National Rice Research Center, Stuttgart, AR, United States; ^2^Adaptive Cropping Systems Laboratory, United States Department of Agriculture, Agricultural Research Service, Beltsville Agricultural Research Center, Beltsville, MD, United States

**Keywords:** inorganic As, alternate wetting and drying, chromosome segment substitution line, soil volumetric water content, rice, quantitative trait loci

## Abstract

Arsenic (As) accumulation in rice grain is a significant public health concern. Inorganic As (iAs) is of particular concern because it has increased toxicity as compared to organic As. Irrigation management practices, such as alternate wetting and drying (AWD), as well as genotypic differences between cultivars, have been shown to influence As accumulation in rice grain. A 2 year field study using a Lemont × TeQing backcross introgression line (TIL) mapping population examined the impact of genotype and AWD severity on iAs grain concentrations. The “Safe”-AWD [35–40% soil volumetric water content (VWC)] treatment did not reduce grain iAs levels, whereas the more severe AWD30 (25–30% VWC) consistently reduced iAs concentrations across all genotypes. The TILs displayed a range of iAs concentrations by genotype, from less than 10 to up to 46 μg kg^–1^ under AWD30 and from 28 to 104 μg kg^–1^ under Safe-AWD. TIL grain iAs concentrations for flood treatments across both years ranged from 26 to 127 μg kg^–1^. Additionally, seven quantitative trait loci (QTLs) were identified in the mapping population associated with grain iAs. A subset of eight TILs and their parents were grown to confirm field-identified grain iAs QTLs in a controlled greenhouse environment. Greenhouse results confirmed the genotypic grain iAs patterns observed in the field; however, iAs concentrations were higher under greenhouse conditions as compared to the field. In the greenhouse, the number of days under AWD was negatively correlated with grain iAs concentrations. Thus, longer drying periods to meet the same soil VWC resulted in lower grain iAs levels. Both the number and combinations of iAs-affecting QTLs significantly impacted grain iAs concentrations. Therefore, identifying more grain iAs-affecting QTLs could be important to inform future breeding efforts for low iAs rice varieties. Our study suggests that coupling AWD practices targeting a soil VWC of less than or equal to 30% coupled with the use of cultivars developed to possess multiple QTLs that negatively regulate grain iAs concentrations will be helpful in mitigating exposure of iAs from rice consumption.

## Introduction

Arsenic (As) exposure from rice consumption is a serious and growing concern as rice is a staple dietary crop for half of the world’s population and is cultivated worldwide. Rice has been identified as a major As exposure route for humans and is often the principal source of As where drinking water As concentrations are low (Kile et al., 2007; Meharg et al., 2009). Consumption of rice containing high levels of As is linked to adverse health impacts including cancer (Zhao et al., 2010; Banerjee et al., 2013).

As is naturally present in soil but can be exacerbated by the use of contaminated irrigation water or anthropogenic sources (e.g., mining activities, use of arsenical pesticides, etc.) (Zhao et al., 2010; Kumarathilaka et al., 2018). Paddy rice accumulates As at a higher rate than other cereal crops (e.g., wheat or barley) for two principal reasons (Williams et al., 2007). First, rice is traditionally grown in flooded fields, which creates anoxic soil conditions leading to As mobilization, in the form of arsenite, in the soil solution (Takahashi et al., 2004; Yamaguchi et al., 2011). Second, arsenite, a form of inorganic As (iAs), is taken up through silicic acid transporters in rice (Ma et al., 2008; Norton et al., 2010). Arsenic accumulates throughout rice plant tissues, but in the grain the predominant forms are organic dimethylarsinic acid (DMA) and inorganic arsenite, a class 1 carcinogen (International Agency for Research on Cancer [IARC], 2004; Williams et al., 2006).

Concern over iAs is increasing in rice markets because of its potentially high concentrations in the grain (Schoof et al., 1999). While both polished and brown rice contain As, brown rice tends to have higher As concentrations because As accumulates in the bran layer (Meharg et al., 2008; Sun et al., 2008). Restrictions have been placed on iAs concentrations in rice by FAO/CODEX, with a limit of 200 μg kg^–1^ for polished rice and 350 μg kg^–1^ for husked (brown) rice products (Codex Alimentarius, 2017), and in the United States, the Food and Drug Administration (FDA) has set a limit of 100 μg kg^–1^ for rice used in infant food products (Gray et al., 2012).

Many studies have shown that rice grain As accumulation can be mitigated by implementing one or more periods of soil drying during the growing season. Alternate wetting and drying (AWD), a water saving irrigation management method, can reduce grain As accumulation; however, the extent of grain As reduction appears to be highly dependent on the severity and timing of the soil dry-down period (Carrijo et al., 2018, 2019; Li et al., 2019). A meta-analysis of AWD studies by Carrijo et al. (2017) found a loss of yield has been observed with too severe of a drying period, whereas AWD cycles that are too mild can fail to reduce grain iAs at all (Carrijo et al., 2017). In practice, it is difficult to impose and hold a uniform soil volumetric water content (VWC) across a non-flooded field and is particularly difficult with unpredictable rain events. Moreover, to protect against yield loss or a reduction in milling quality, rice farmers will implement “Safe”-AWD practices, which try to minimize crop stress while maximizing irrigation savings (Carrijo et al., 2018). Thus, it is important to develop rice varieties that have low As accumulation in the grain and also perform well with reduced irrigation practices.

Multiple studies have established that rice cultivars vary in As accumulation rates; however, the genetics that influence accumulation or exclusion of As are not as well understood. Using mapping populations such as chromosome segment substitution lines (CSSLs) to study grain As accumulation can facilitate identification of As-affecting quantitative trait loci (QTLs). Previous studies have identified QTLs for total As accumulation in shoots and roots after applying an iAs treatment (Murugaiyan et al., 2019), as well as total As (Dasgupta et al., 2004; Zhang et al., 2007, 2014; Norton et al., 2010, 2012, 2014) and organic As accumulation (DMA) in the grain (Kuramata et al., 2013). However, identification of QTLs specifically affecting grain iAs, as opposed to total or organic As fractions, has not been previously reported in the literature. Identifying QTLs affecting iAs accumulation in rice grain can inform breeding efforts toward developing low iAs accumulating varieties. Additionally, understanding how the combined effect of irrigation management practices and QTL presence impact the accumulation of grain iAs is important for producing low As cultivars. The objective of this research is threefold: to identify genomic regions (i.e., QTLs) controlling iAs accumulation in rice grain, to understand how specific QTLs and their combinations influence grain iAs concentrations, and to understand changes in grain iAs levels resulting from QTL combinations in response to irrigation management practices. Ultimately, the goal of this research is to inform future marker-assisted breeding toward As excluding varieties to mitigate human exposure to As from rice consumption.

## Materials and Methods

### Field Experiment

A set of 123 CSSLs, developed from backcrossing “Lemont” (tropical Japonica) as the recurrent parent and “Teqing” (*indica*) as the donor as described by Pinson et al. (2012), was used in this study. Seeds were obtained from the USDA-ARS Genetic Stocks Oryza (GSOR) collection^1^. The experiment was conducted at the Dale Bumpers National Rice Research Center in Stuttgart, AR, during the summer of 2013 and 2014. The soil at the site is described as a Dewitt silt loam (fine, smectitic, thermic, Typic Albaqualfs) with a total C content of 0.67% and a total N content of 0.085%. The naturally occurring As content of this soil has previously been reported to be between 5.5 and 6.8 mg kg^–1^ (Somenahally et al., 2011a,b). A soil analysis was performed prior to planting each year followed by 100 and 56 kg ha^–1^ phosphorous (P_2_0_5_) applied in 2013 and 2014, respectively, along with 100 kg ha^–1^ potassium (K_2_O) and 11 kg ha^–1^ zinc. Pre-emergent herbicide was applied, and additional pesticide treatments were applied as needed throughout the growing season according to local recommendations. The study consisted of 117 and 121 TeQing-into-Lemont backcross introgression lines (TILs) (genotypes) in 2013 and 2014, respectively, along with six repeated check plots of the two parents, evaluated under two irrigation regimes. Each irrigation treatment was conducted in separate adjacent fields. Within each irrigation regime, the TILs were arranged as a randomized complete-block design with four replications; however, only one field replication was used for the grain As analysis (see below).

The studies were planted on May 30, 2013, and June 5, 2014, and the TILs were sown in hill plots with a Hege 1000 plot planter (Wintersteiger Inc., Salt Lake City, UT), with each experimental unit consisting of 10 plants having approximately 30 cm between plants. All fields were irrigated after planting to ensure uniform germination and stand establishment. Approximately 2 weeks after emergence, 56 kg N ha^–1^ was applied as urea, followed by flood irrigation. For the remainder of the season, the flood treatment was sustained, whereas the AWD treatment was allowed to dry until the target soil VWC was reached (see below for VWC targets). The field was then flooded for 3 days, drained, and allowed to dry to the target VWC again; cycles were repeated a total of four times during the season (Table 1). A POGO Pro (Stevens Water Monitoring Systems Inc., Portland, OR, United States) was used to monitor soil moisture at a depth of 5 cm on about half of the experimental units to determine when to irrigate the AWD treatment. In 2013, a Safe-AWD treatment was implemented with a target soil VWC range of 35–40%. No plant stress symptoms were observed in 2013; thus, in 2014, the severity of the AWD cycles was increased to a target range of 25–30% VWC (hereafter referred to as AWD30) (Table 1). Soil VWC measurements are reported for the specific experimental units analyzed for grain iAs in this study. Temperature and rain data were collected on site by the USDA-ARS weather station^2^.

**TABLE 1:** Severity of alternate wetting and drying (AWD) treatment and mean soil volumetric water content (VWC, %) at the end of each AWD cycle by experiment.

Site	Year	AWD severity	Grow location	Mean soil VWC (%) at the end of AWD			
				1st AWD	2nd AWD	3rd AWD	4th AWD
Dale Bumpers National Rice Research Center, Stuttgart, AR	2013	Safe-AWD	Field	43	40	34	44
	2014	AWD30	Field	37	25	28	23
Beltsville Agricultural Research Center, Beltsville, MD	2019	AWD20	Greenhouse 1	20	20	—	—
			Greenhouse 2	19	20	—	—

Two plants per experimental unit were monitored for days to heading and were harvested individually at maturity for collection of all agronomic data for each of the four field replications. The plants were dried to approximately 12% moisture, threshed individually, and agronomic traits were determined as an average of the two plants per experimental unit, across the four field replications. The paddy rice samples were stored at 4°C prior to further analysis.

### Analysis of Grain iAs

Rough rice from one of the harvested plants for each experimental unit in one field replication per irrigation treatment was shelled using the Testing Husker (Satake Engineering Co., Uino Taito-Ku, Tokyo) to produce between 3 and 10 g of whole-grain brown rice in 2013 and 2014. Because of low yields, in some cases both of the plants per experimental unit were combined prior to shelling to reach a minimum of 3 g of brown rice per genotype. The grain samples were ground to a fine powder using a UDY Cyclone Sample Mill (Fort Collins, CO, United States) with a 0.8 mm screen. Ground samples were stored at 4°C for later analysis. iAs was measured following the protocol described in Chaney et al. (2018); all reagents were trace metal grade. Briefly, 0.7 g (dry weight) rice flour was weighed into 50 mL polyethylene tubes. Samples were extracted using the standard hot block method (Gray et al., 2012) with 10 mL of 0.28 M HNO_3_ for 90 min at 95°C. At the end of extraction, tubes were removed from the hot block and allowed to reach room temperature before filtering the extracts through Whatman Grade 40 filter paper into new 50 mL polyethylene tubes. Filters were rinsed twice with 0.28 M HNO_3_, and the filtrate was diluted to 20 mL with 0.28 M HNO_3_.

iAs in filtered extracts was measured by inductively coupled plasma atomic emission spectroscopy using a hydride generation flow injection method (Musil et al., 2014; Pétursdóttir et al., 2014). Technical replicates of the NIST (National Institute of Standards and Technology) standard reference for rice flour 1568b (certified values for total As of 285 ± 14 μg kg^–1^, iAs of 92 ± 10 μg kg^–1^, DMA of 180 ± 12 μg kg^–1^ and monomethylarsonic acid of 11.6 ± 3.5 μg kg^–1^) and in-house standards were run for quality assurance. Additionally, control blank, spiked samples, and random technical replicates of samples were also analyzed. In this study, the mean measured iAs for the NIST 1568b standard was 99 ± 2 μg kg^–1^, which is within 1 standard deviation of the certified value of 92 ± 10 μg kg^–1^.

### QTL Analysis and TIL Selection

Grain iAs results from the 2013 and 2014 field study of the TILs and their parental lines were used in QTL mapping using IciMapping 4.1^3^ (Meng et al., 2015). Genotypic data were obtained from next-generation sequencing according to Elshire et al. (2011) and processed using the Tassel 5 GBS v2 Pipeline (Glaubitz et al., 2014). The stepwise regression method (RSTEP-LTR) of IciMapping was used to identify QTLs. The threshold for the log-of-odds to declare significant QTLs was calculated via a 1,000 permutation test to ensure genome-wide type 1 error rate to be ≤ 0.05. The identified QTLs and grain iAs concentrations were used to select eight TILs from the mapping population. The selected TILs represent a span of low to high grain iAs accumulating phenotypes and differ in number and combinations of the seven identified QTLs. Additionally, the subset was selected to have heading days within 2 weeks to ensure developmental stages were similar within the selected set. Four genotypes were selected to represent each of the grain iAs excluder and grain iAs accumulator groups.

### Greenhouse Study

In 2019, the eight selected TILs and their parents (Lemont and TeQing) were grown concurrently in two adjacent greenhouses at the USDA-ARS, Beltsville Area Research Center in Beltsville, MD. Sterilized seeds were germinated and then planted in 72-cell trays in potting soil. Approximately 2 weeks later, a single seedling was transplanted to a 19-l bucket made of high-density polyethylene filled with soil collected from the plow layer of a field used in the 2013/2014 field study at the Dale Bumpers National Rice Research Center in Stuttgart, AR. The buckets were filled with soil to a depth of 27 cm, leaving approximately 10 cm above the soil surface to allow for watering.

The eight TILs and two parents were grown in four biological replicates per greenhouse and evaluated using two water treatments (10 genotypes × 4 replications × 2 water treatments × 2 greenhouses). Pots were arranged in two randomized complete blocks per bench, with four benches per greenhouse. All pots on a bench were subjected to the same water treatment and treatments alternated by bench (e.g., AWD on bench 1, flood on bench 2, etc.), for a total of two AWD and two flood benches per greenhouse. Benches in the two greenhouses differed in distance from the cooling pad, and for this reason, the order of treatments alternated between the greenhouses (e.g., bench 1 nearest the cooling pad was AWD in greenhouse 1 but was flood in greenhouse 2).

Two water management treatments were used: flood (continuously flooded control) and AWD during which two cycles of wetting and drying were completed (Table 1). The target soil VWC for drying was 20% (AWD20) in 2019, which was more severe than that used in the field studies. Beginning 2 weeks after transplanting, all pots were grown under flooded conditions until the AWD treatment was started approximately 4 weeks prior to panicle initiation. Flooding was held at a water depth of approximately 10 cm above the soil surface. Urea (80 kg N ha^–1^) was applied as N fertilizer in a three-way split during the 2 months following transplanting.

Soil VWC was monitored continuously using TDR probes (CS650 s from Campbell Scientific, Logan, Utah; and Acclima 310 s from Acclima, Meridian, Idaho) for each individual pot in the AWD treatment. Probes were installed to measure soil VWC at a depth of 0–10 cm. Soil VWC was monitored in one flood control pot per greenhouse. For AWD treatments, pots were drained, and when the soil in a pot reached the target VWC (± 1%), it was reflooded. Thus, each pot was reflooded based on their individually measured VWC, and reflooding dates varied by pot, which is in contrast to field conditions where average field VWC was used to decide when to reflood the entire field. The flooded state was held for 8–10 days to ensure soil water saturation before draining and starting the second AWD cycle. The two AWD cycles were completed prior to heading stage after which pots were reflooded until harvest. Soil samples from the greenhouse were also analyzed for total As. The samples were analyzed using the Niton XL3t XRF Analyzer (Thermo Fisher Scientific, Waltham, MA, United States) and measured 7.1 ± 1.3 mg kg^–1^.

Plants were harvested at maturity, and grain samples for each biological replicate (*n* = 8 across the two greenhouses) were processed for iAs analysis as described above. Additional agronomic traits including tiller and mature panicle number, shoot biomass without panicles, and total grain yield were determined for each plant at harvest. Temperature, humidity, and light measurements were also recorded for each greenhouse.

### Statistical Analysis

All data analyses were conducted in R (version 3.6.1) (R Core Team, 2013) and IciMapping 4.1^4^. Differences in grain iAs accumulation between genotypes or number of QTLs were determined using analysis of variance (ANOVA) with a *p* < 0.05 and using Tukey HSD for multiple mean comparisons using a *p* < 0.05. Individual genotype comparisons between treatments (flood and AWD) were tested for significance using Welch *t*-tests.

## Results

### Field Study 2013 and 2014 Grain iAs

In 2013, the targeted average soil VWC was achieved at 40%, which is near saturation (termed Safe-AWD) for this soil type. There was little rainfall prior to heading, which occurred during the week prior to the fourth AWD cycle, but more rainfall occurred during the later grain fill stage (Supplementary Figure 1A). Because no plant stress was observed in 2013, the goal in 2014 was to decrease the soil VWC to a target of 25–30%. At an average of 28.3% across the four AWD cycles, this target was achieved in 2014 despite the more frequent rainfall observed prior to heading, which occurred during the week following the fourth AWD cycle (Supplementary Figure 1B).

The frequency distribution of brown rice iAs concentrations varied by water treatment and year (Supplementary Figure 2). In 2013, the AWD was less severe (Safe-AWD) than in 2014 (AWD30), resulting in little to no difference in grain iAs accumulation between AWD and flood treatments. In 2014, the more severe drying cycles resulted in a marked decrease in grain iAs by AWD as compared to flood for the TILs and both parents.

### QTL Identification and Impact on Grain iAs Levels

**FIGURE 1:**
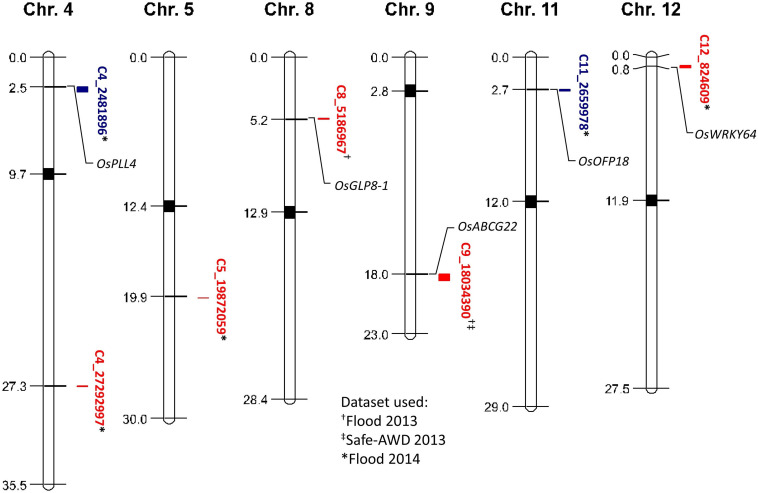
Map of quantitative trait loci (QTLs) affecting brown rice grain inorganic As concentrations and candidate gene locations. Red and blue indicate positive and negative QTL effects on grain iAs levels, respectively. Datasets used to identify QTLs are shown with symbols following the QTL identifier, flood 2013 (^†^), Safe-AWD 2013 (^‡^), flood 2014 (^∗^). Approximate locations of genes colocalized with the identified iAs affecting QTLs are depicted with italicized gene names.

No QTLs were identified by irrigation treatment when data were pooled across years because of the large environmental variation between the 2013 and 2014 field seasons. Thus, data were grouped by each year and treatment combination for QTL identification. Seven QTLs affecting grain iAs accumulation were identified in the TIL mapping population corresponding to chromosomes 4, 5, 8, 9, 11, and 12 (Figure 1, detailed information on QTLs in Supplementary Table 1). Two QTLs affecting grain iAs were identified from the 2013 flood treatment, five QTLs were identified from the 2014 flood treatment, and one QTL from the 2013 Safe-AWD was also identified from the 2013 flood condition (C9_18034390). Two TeQing introgression QTLs were associated with decreased grain iAs levels (C4_2481896 and C11_2659978), whereas the other five QTLs (C4_27292997, C5_19872059, C8_5186967, C9_18034390, C12_824609) were associated with increased grain iAs levels. No genotype-by-management (G × M) QTLs affecting iAs levels were found.

**FIGURE 2:**
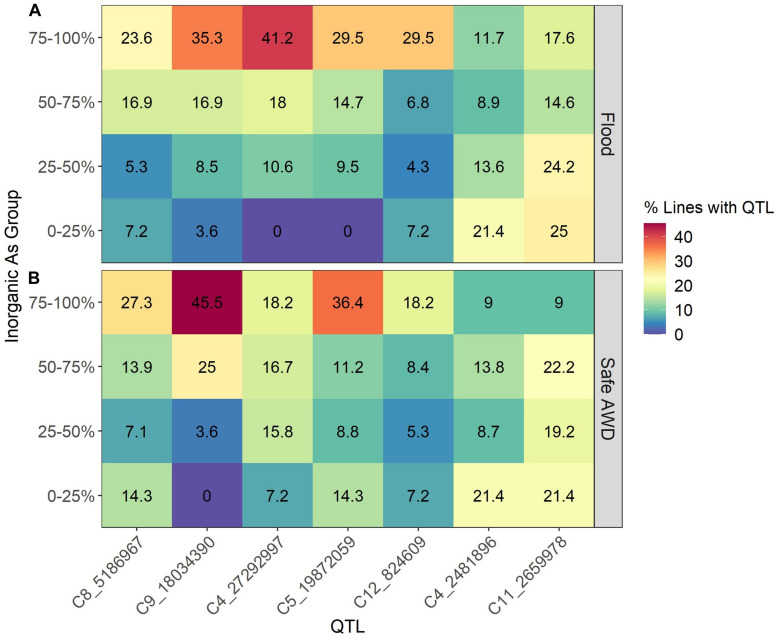
Percent of lines containing each of the identified QTLs among groups based on the range of inorganic arsenic observed in each experiment (e.g., 0–25% represent those samples whose concentrations are < 25% of the maximum observed inorganic As for that experiment). **(A)** Flood 2013 and 2014; **(B)** Safe-AWD in 2013.

To examine which of the identified QTLs are most abundant in the TILs, we calculated the percent of lines containing each QTL among four grain iAs groups defined by different ranges in iAs concentrations (Figure 2). Groups were determined for the two treatment-year datasets (Figure 2A, flood 2013 and 2014; Figure 2B, Safe-AWD in 2013) by ranking the measured grain iAs concentrations and dividing the resulting range into four groups where each group represents a quarter of the full range of concentrations (e.g., 0–25% represent those samples whose concentrations are less than 25% of the maximum observed iAs for that experiment). Within each iAs group, we calculated the percent of genotypes containing each identified QTL. In general, the genotypes in the top 75% of grain iAs concentrations had a higher percentage of each of the five QTLs identified that are associated with increased iAs (C4_27292997, C5_19872059, C8_5186967, C9_18034390, C12_824609) when compared to the other lower iAs groups. Furthermore, the two QTLs (C4_2481896 and C11_2659978) having the opposite effect were more abundant in the low iAs group. The high iAs concentration group (75–100%) tends to have a higher number of positive iAs-associated QTLs and is more likely to have C4_27292997, C5_19872059, and C9_18034390 than those in the lower iAs groups.

To examine whether the presence of other TeQing background introgressions, not specifically identified as iAs affecting QTLs, was influencing the observed grain iAs concentrations, the effect of TeQing kinship (i.e., the fraction of a TIL’s genome coming from TeQing introgressions) was explored. However, there was no correlation between iAs accumulation and how closely related a TIL is to TeQing (Supplementary Figure 3). Thus, there is no intrinsic bias in the TIL population as a whole for grain iAs as a result of TeQing background introgressions.

### QTL Impact on Grain iAs Accumulation

**FIGURE 3:**
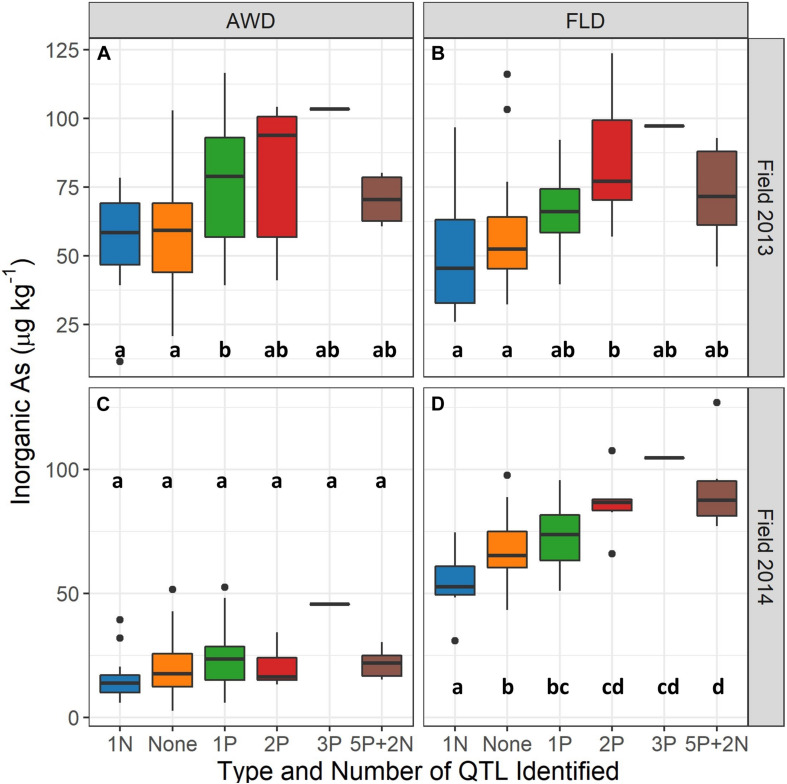
Brown rice inorganic As concentrations (μg kg^– 1^) in field 2013 under alternate wetting and drying (AWD) **(A)** and flooded (FLD) **(B)** and 2014 AWD **(C)** and flood **(D)** irrigation management treatments by number of quantitative trait loci (QTLs) in eight TILs and two parents. Safe-AWD was used in 2013, and AWD30 was used in 2014. Only one biological replication of the genotype containing three positive iAs affecting QTLs was grown in 2013 and 2014; all other QTL groups have a minimum of six biological replicates. Letters indicate significant differences within each treatment and year (*p* < 0.05).

Grain iAs levels increase concomitantly with an increasing number of identified QTLs in both the AWD and flood field management treatments across the entire mapping population (Supplementary Figure 4), which was stronger under flood conditions than AWD conditions. The eight TILs and two parents grown across all experiments (see *TIL Selection for Greenhouse Study*) were examined separately, and a similar pattern was observed where grain iAs levels increased with increasing number of QTLs identified. The relationship was again more apparent under flood and the 2013 Safe-AWD treatments as compared to the more severe 2014 AWD30 (Figure 3).

### TIL Selection for Greenhouse Study

The eight TILs selected based on iAs grain concentrations and number and combination of QTLs represent a set of low and high As accumulators (Table 2), which may allow for further investigation of genetic influence on grain iAs accumulation. Both TIL596.11 and TIL643 have no identified grain iAs QTLs and were chosen for comparison to Lemont as all TILs in the population have some level of random background TeQing introgressions (Pinson et al., 2012).

**TABLE 2:** Genotypes selected for the greenhouse study including parents, Lemont and TeQing, and eight TILs.

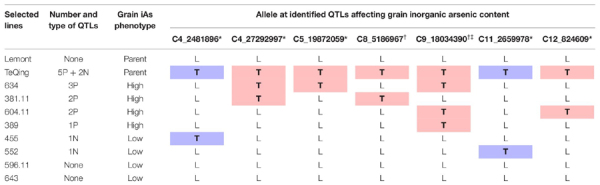

*Datasets used to identify QTLs are denoted by symbols: ^†^Flood 2013, ^‡^Safe-AWD 2013, ^∗^Flood 2014. The number and type of identified QTLs are shown for each line with the notation chromosome number, followed by position in bp (e.g., chromosome 8 position 5,186,967 bp is C8_5186967), with L and T representing Lemont or TeQing alleles. Letters P and N indicate the number of TeQing QTL introgressions that are either positively (P) or negatively (N) affecting grain iAs. Letters within a QTL column indicate the presence of the TeQing introgressed allele (T) or the Lemont recurrent parent allele (L). Red and blue highlights indicate the TeQing allele increases or decreases grain inorganic arsenic, respectively. No highlight indicates Lemont was the segment present in the identified QTL region.*

### Greenhouse 2019 Grain iAs Accumulation and QTL Impact

**FIGURE 4:**
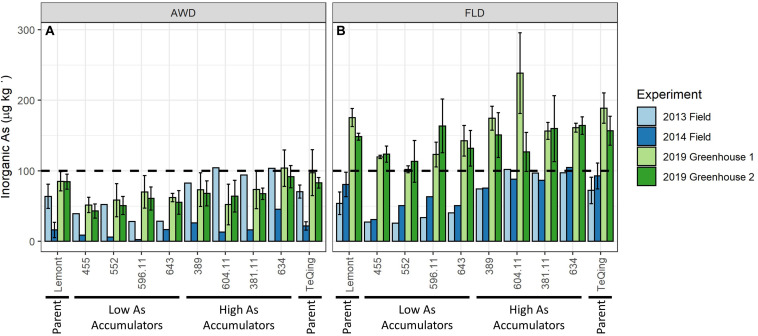
Comparison of brown rice inorganic arsenic concentrations for eight TILs and their parents. Genotypes were grown in the field in 2013 and 2014 and in two greenhouse studies (Greenhouse 1 and Greenhouse 2) in 2019 under alternate wetting and drying (AWD) **(A)** and flooded **(B)** irrigation management practices. Error bars represent standard deviation of biological replicates (*n* = 4 in 2019 for each greenhouse, and *n* = 6 in 2013 and 2014 for parents only). Dotted line represents FDA limit of 100 μg kg^– 1^ for inorganic arsenic in rice-based infant food.

The eight selected TILs and their parents grown in the greenhouse (greenhouse environmental conditions are summarized in Supplementary Figure 5) showed significant (*p* < 0.05 for all) reductions in grain iAs accumulation under AWD20 as compared to flood management practices (Figure 4 and Table 3). Indeed, most TILs with the exception of two high iAs accumulators (TIL 634 and TeQing) meet the FDA limit of 100 μg kg^–1^ when grown under AWD20 practice with only 2 dry down cycles completed before heading stage. Analysis of variance indicated that there was no significant genotype × irrigation treatment effect on any trait measured (data not shown).

**TABLE 3:** Percent change in brown rice inorganic arsenic of eight TILs and their parents from AWD to flood treatments in studies conducted in the field and greenhouse.

Type	Name	Observed grain iAs phenotype	Percent change in grain inorganic As between AWD to flood treatments				
			Field 2013	Field 2014	Greenhouse 1	Greenhouse 2	Average of greenhouse studies
Parent	Lemont	Parent	**+ 18**	−80*	**−51.5**	**−42.9**	**−47.2**
TIL	455	Low	+ *43*	*−71.2*	**−56.9**	**−65.3**	**−61.1**
TIL	552	Low	+ *101*	*−88.2*	**−42.7**	**−55.2**	**−48.9**
TIL	596.11	Low	*−16.7*	*−95.9*	**−43.0**	**−62.9**	**−53.0**
TIL	643	Low	*−29.6*	*−67.4*	**−56.5**	**−58.1**	**−57.3**
TIL	389	High	+ *10.9*	*−65.4*	**−58.0**	**−54.8**	**−56.4**
TIL	604.11	High	+ *2.3*	*−85.0*	**−78.1**	**−49.5**	**−63.8**
TIL	381.11	High	*−3*	*−81.4*	**−53.1**	**−57.8**	**−55.4**
TIL	634	High	+ *6.5*	*−56.5*	**−35.5**	**−44.2**	**−39.9**
Parent	TeQing	Parent	**−2.3**	−76.6*	**−48.5**	**−47.0**	**−47.7**

Percent change values in bold font indicate significant differences in mean inorganic As levels between flood and AWD treatments based on Welch t-test (p < 0.05). Italics indicate no test of significance was performed because of lack of replication. Biological replicates were used in calculations, n = 4 in 2019 for each greenhouse, n = 6 in 2013 and 2014 for parents Lemont and TeQing, and n = 1 for the TILs in 2013 and 2014). Asterisk (*) indicates significant difference based on Welch t-test (p < 0.05) for grain inorganic As concentrations between field 2014 and average greenhouse 2019 results.

Containing any single QTL alone did not appear to impact differences in grain iAs accumulation in the greenhouse study (Supplementary Figure 6) as much as the number and type (i.e., positive or negative) of QTL contained by the genotype (Supplementary Figure 7). TILs containing QTLs negatively affecting iAs levels in the 2013 and 2014 field study (C11_265997 or C4_248189) represent the lowest mean grain iAs concentrations in the greenhouse data (Figure 3 and Supplementary Figure 7).

### Comparison of Field vs. Greenhouse Studies

There is a noticeable increase in grain iAs concentrations in greenhouse-grown rice as compared to field-grown rice, particularly under the flooded treatment (Figure 4). Lack of replication in the field study for grain iAs prohibits any statistical comparisons. However, there was replication of the parents, Lemont and TeQing, which show a statistically significant higher grain iAs accumulation between the 2014 field and the 2019 greenhouse results under flooded (*p* < 0.001, Table 3).

Genotype differences in mean grain iAs levels within treatments were also examined, and some were found to be significant under flood (greenhouse 1) and AWD (greenhouse 2) (data not shown); however, the most significant differences were observed between the AWD and flood treatments. To compare iAs reductions between treatments, we calculated the percent change in grain iAs accumulation between flood and AWD in all studies (Table 3). The four cycles of Safe-AWD used in 2013 were not effective in reducing grain iAs (ranging from a reduction of 30% to an increase of 101%), whereas the more severe AWD30 used in 2014 did result in consistent reductions across all eight TILs and their parents (ranging from a reduction of 56–96%). Similarly, in 2019, under the more severe two-cycle AWD20 in the greenhouse study, all TILs and their parents had significantly reduced iAs in the rice grain (Table 3). In 2019, reductions by AWD in grain iAs were generally 40% or greater (*p* < 0.05 for both greenhouses) across almost all genotypes, with TIL634, a high iAs accumulator, in greenhouse 1, the only exception having a reduction of 36% in grain iAs concentrations under AWD. TIL634 had some of the lowest reductions in grain iAs from flood to AWD treatments overall. In contrast, the low iAs accumulator TIL455 had some of the greatest iAs reductions from flood to AWD with a mean reduction across both greenhouses in 2019 of 61%. In 2014 under AWD30, four TILs, 552, 596.11, 604.11, and 381.1, reduced grain iAs accumulation by at least 80%, whereas under the more severe AWD20 in 2019, the same TILs on average reduced iAs by markedly less, ranging from 49 to 64% on average across two greenhouses.

### Impact of Days Under AWD on Grain iAs Accumulation

**FIGURE 5:**
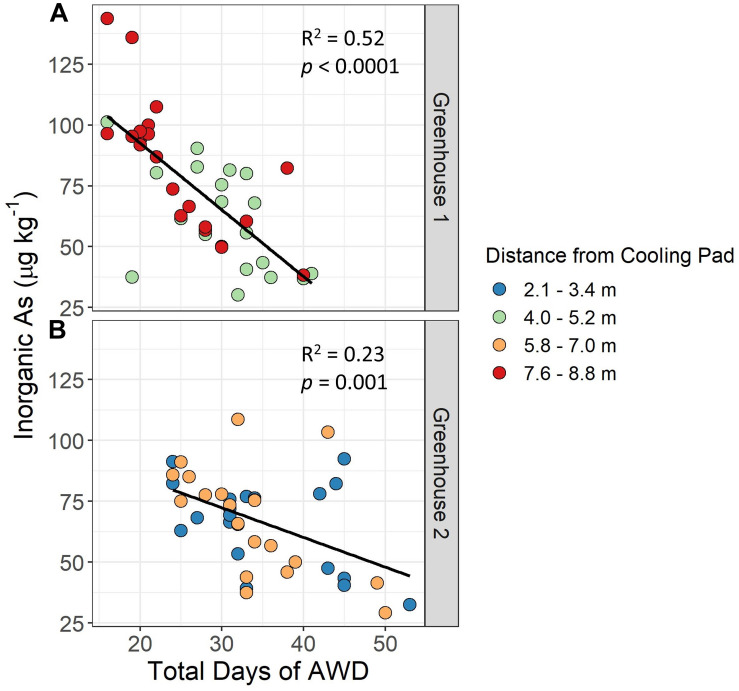
Impact of days under AWD on grain inorganic As levels. Brown rice inorganic As accumulation in response to the total number of days to achieve the target soil volumetric water content (VWC) using alternate wetting and drying (AWD) cycles (e.g., sum of the total days under AWD for cycles 1 and 2) and distance of experimental units from the greenhouse cooling pad. In greenhouse 1 **(A)** the AWD tables were farther from the cooling pad than in greenhouse 2 **(B)**.

In the greenhouse study, grain iAs accumulation was significantly negatively correlated to the total number of days a plant was under AWD to achieve the target soil VWC of 20% (i.e., the sum of days under AWD for the first and second AWD cycles) (Figure 5). In general, plants in greenhouse 1 had faster AWD drying cycles as compared to plants in greenhouse 2. Total days of AWD under the faster AWD of greenhouse 1 ranged from 16 to 41 days vs. 24 to 53 days under the slower AWD in greenhouse 2. Thus, it took approximately 10 more days under the slow AWD to achieve the same target soil VWC as the fast AWD. Additionally, the grain iAs levels were reduced to a greater extent under slow AWD as compared to fast AWD. The range of grain iAs levels under fast AWD was 32–138 μg kg^–1^ as compared to 31–110 μg kg^–1^ under slow AWD.

Pot distance from the cooling pad (Supplementary Figure 8) influenced the soil drying time. Because greenhouse cooling pads created a temperature gradient in each greenhouse, we chose to alternate AWD and flood treatment benches by greenhouse. In greenhouse 1, a flood treatment was imposed on the first bench nearest the cooling pad, whereas in greenhouse 2, the first bench nearest the cooling pad was an AWD treatment. The greenhouse temperature gradient affected drying time by pot distance to the cooling pad. In greenhouse 1 the plants that were farther from the cooling pad (7.6–8.8 m) dried faster than those closer to the cooling pad (4.0–5.2 m), with a similar trend apparent in greenhouse 2. This suggests there was a temperature gradient in the greenhouses, and the gradient in greenhouse 1 was stronger than that of greenhouse 2, which ultimately affected the total days under AWD.

### Agronomic Traits

In general, there was a decrease in grain yield under the more severe AWD30 and AWD20. However, only 1 of the 10 genotypes had a statistically significant decrease in total yield due to irrigation treatment; TIL 381.11 in greenhouse 2 had a 30% reduction (*p* = 0.029) in yield under AWD as compared to flood (Supplementary Table 2).

In the greenhouse study, there was a trend for genotypes with increasing number of As accumulating QTLs to have higher mean total yield (Supplementary Figure 9), mature panicle number (Supplementary Figure 10), and tiller number (Supplementary Figure 11); however, this increase was only significant for mature panicle number and total yield (*p* < 0.05).

**FIGURE 6:**
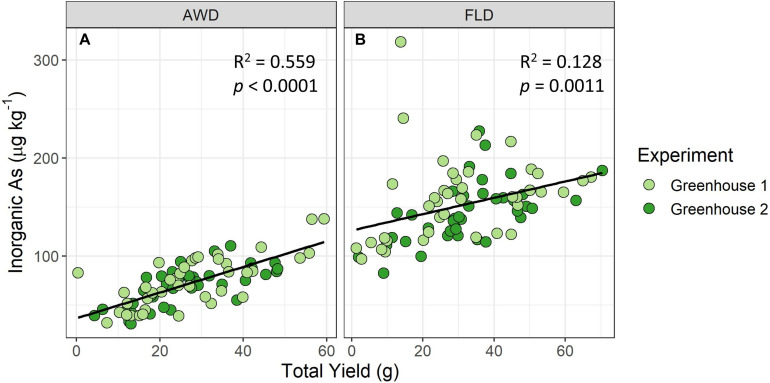
Brown rice inorganic As accumulation vs. total grain yield in greenhouse experiment under AWD20 (AWD) **(A)** and flood (FLD) **(B)** treatments. A significant positive correlation is seen between total grain yield and grain inorganic As concentrations in the greenhouses.

A significant, strong correlation was found between grain iAs accumulation and total yield in the greenhouse AWD treatment (Figure 6) but was only significant and moderately strong in the field for the 2014 flood treatment (Supplementary Figure 12). Mature panicle number had no clear relationship with grain iAs concentrations (Supplementary Figure 13), and no strong correlations were found between tiller number (Supplementary Figure 14) or days to heading (Supplementary Figure 15) and grain iAs accumulation from the field experiments.

## Discussion

### Inorganic As Reductions by AWD

Mitigation of As accumulation in rice is dependent on the severity of AWD cycles. Several studies have shown that the more severe the AWD, the greater the reduction in both total As (Hu et al., 2013; Linquist et al., 2015) and iAs in rice grain (Carrijo et al., 2019; Li et al., 2019). In 2013, the Safe-AWD with a target soil VWC of 35–40% was not severe enough to reduce iAs concentrations. In fact, in this study, iAs was the same or even increased for some TILs under the Safe-AWD in 2013 as compared to flood, whereas in 2014, the more severe AWD30 led to consistent reductions in grain iAs across all TILs. These results are similar to those reported by Carrijo et al. (2019). In our field study, it is likely that the soil did not become fully aerobic during the Safe-AWD of 2013 as the soil VWC did not drop sufficiently below saturation (saturated soil VWC in the field is slightly above 40%). Thus, microbial processes that oxidize As making it less bioavailable could not occur, allowing As to be present in the soil solution as the reduced arsenite form, which is easily taken up by rice (Ma et al., 2008; Norton et al., 2010; Zhao et al., 2010). In comparison, in both the field 2014 and the greenhouse study, the imposed AWD cycles were severe enough to make the soil fully aerobic, allowing for geochemical and microbial processes to oxidize As, resulting in sequestration into iron and manganese oxides (Takahashi et al., 2004; Zecchin et al., 2017; Maguffin et al., 2020).

The AWD implemented in the greenhouse pots was more severe (20% VWC) and controlled than that realized in the field (30–40% VWC). Additionally, the measurement depth for soil VWC in the greenhouse (10 cm) as compared to the field (5 cm) likely compounded the difference in severity of the AWD cycles experienced by the plants in the greenhouse vs. those in the field. In fact, Maguffin et al. (2020) demonstrated that even when severe AWD cycles were implemented in field soil at the Stuttgart, Arkansas, location and low soil VWC was achieved at soil depths of 10 cm, there was only a small decrease in soil moisture at the 25 cm depth. Because a shallower depth was used for VWC measurements in the field, it was assumed that the AWD cycles would be even less severe in the field compared to those in the greenhouse. However, although only two cycles of AWD20 were used in the greenhouse, the net reductions in grain iAs were significant but lower in magnitude than those observed with the four-cycle AWD30 used in the field (mean reduction under AWD20 in the greenhouse was 53% as compared to 77% for AWD30 in the field). When averaged over the two greenhouse experiments, the two-cycle AWD20 effectively reduced iAs across the 10 genotypes with a minimum grain iAs reduction of 40% from flood to AWD (Table 3). The consistent reduction in grain iAs across the years regardless of severity in the field (AWD30) or in the greenhouse (AWD20) is evident; however, future studies implementing AWD in the field will need to monitor VWC levels at a greater soil depth to be comparable to the AWD treatment achieved in this greenhouse study.

AWD severity is the driving factor reducing grain As; however, the timing of AWD cycles is an important factor for yield and may also play a role in grain As reduction. A handful of studies have examined how the number and timing of AWD cycles impact total and iAs reduction. A study by Linquist et al. (2015) found that one AWD cycle followed by sustained flooding after plants reached the reproductive stage resulted in higher total As concentrations as compared to those that underwent repeated AWD cycles of the same severity (Linquist et al., 2015). This suggests the success of AWD in reducing grain total As accumulation is tied to both the number and, importantly, the timing of AWD cycles. However, reductions in grain total As due to AWD do not necessarily result in a concomitant reduction in grain iAs. Indeed, several studies have found low severity AWD can reduce total As by reducing the organic As fraction, while the inorganic fraction remains unchanged, whereas a more severe AWD or aerobic growth can reduce both (Xu et al., 2008; Carrijo et al., 2019; Li et al., 2019). Carrijo et al. (2019) found that severe soil drying late in the growing season (either at booting or heading stage) was needed to decrease iAs concentration in rice grain compared to flooded treatments (Carrijo et al., 2019). In our greenhouse study, the first AWD cycle began prior to panicle initiation, and the second AWD cycle was completed around the onset of heading stage; all cycles were completed before grain fill. In 2014, the AWD cycles began earlier in the plants’ development and were ongoing during booting stage, but the majority of plants were heading after the fourth AWD cycle. Thus, optimal timing for completion of AWD cycles appears to be before plants reach the grain fill stage. In our study, we cannot decouple the severity of AWD from the timing of AWD; future studies should focus on imposing moderate to severe AWD cycles before plants reach the grain fill stage to confirm the trend observed here.

Interestingly, we found a significant correlation (*p* < 0.0001 for greenhouse 1 and *p* = 0.001 for greenhouse 2) between the number of days under AWD and grain iAs accumulation, even though all plants were subjected to the same AWD severity. This relationship was stronger in greenhouse 1 where the AWD was faster (*R*^2^ = 0.52) compared to greenhouse 2 (*R*^2^ = 0.23) where the AWD was slower, likely in part because the AWD tables in greenhouse 1 were farther from the cooling pad than in greenhouse 2 (Supplementary Figure 8), leading to shorter drying cycles. The correlation between days under AWD and iAs accumulation suggests that the duration of the AWD cycle could be as important as the severity of drying. It is possible that a less severe AWD held for a longer period may reduce grain iAs concentrations more than a relatively fast AWD cycle targeting the same water stress level. Under drying conditions, as the soil VWC drops, the soil redox potential concomitantly begins to increase, creating favorable conditions for iAs to be oxidized and adsorbed to iron and manganese oxides (Maguffin et al., 2020). We measured redox potential in a subset of pots in the greenhouses and confirmed that those under flooded conditions had low redox potential (approximately -400 mV), and as they dried, their redox potential increased rapidly (up to approximately +375 mV) (Fernández-Baca et al., in preparation). This indicates conditions in the pots became favorable for iAs oxidation, likely leading to iAs adsorption, reducing its bioavailability. Previous studies have shown that rice grown under aerobic conditions has lower grain iAs concentrations (Xu et al., 2008). However, aerobic growth can negatively impact yield, whereas multiple studies have shown that AWD can reduce grain iAs without decreasing yield (Das et al., 2016; Honma et al., 2016; Yang et al., 2019), and there is evidence it may even increase yield (Norton et al., 2017). Indeed, Honma et al. (2016) found that increasing the number of AWD drying days from 1 to 5 days significantly reduced grain iAs by 60% with no reduction in yield. Similarly, we found increased days under AWD reduced grain iAs (Figure 5), and only one genotype (TIL381.11) had a significant reduction in yield under AWD (Supplementary Table 2).

### Genotype and Environmental Impact on iAs Accumulation

A number of studies have shown that rice genotypic differences influence grain As accumulation and speciation (Williams et al., 2005; Zavala et al., 2008; Jia et al., 2014). Williams et al. (2005) measured total As, organic As, and iAs in 36 rice varieties grown in pots and found that the proportion of iAs in the grain is significantly dependent on the rice genotype (Williams et al., 2005). Our study focused on iAs because of its higher toxicity to humans compared to organic As (Schoof et al., 1999; International Agency for Research on Cancer [IARC], 2004) and evaluated brown rice samples where iAs is concentrated in the bran (Meharg et al., 2008; Sun et al., 2008). We found genotypic differences in grain iAs contents among a large set of CSSLs, which ranged from less than 10 up to 46 μg kg^–1^ under AWD30 and 28 up to 104 μg kg^–1^ under Safe-AWD (Supplementary Figure 2). Concentrations for flood treatment in the field study ranged from 31 to 127 μg kg^–1^ in 2013 and 26 to 124 μg kg^–1^ in 2014 (Supplementary Figure 2). Within the CSSL mapping population, some TIL offspring displayed more extreme (i.e., higher or lower) iAs grain phenotypes than either parent, regardless of treatment (Supplementary Figure 2). This is characteristic of transgressive segregation in which offspring display a phenotype outside of the parental range. Indeed, transgressive phenotypes are more likely to occur when two parents have a wider genetic diversity as in our study (de los Reyes, 2019). In our greenhouse study, the transgressive phenotype was most apparent when comparing the low iAs accumulator TIL455 (mean grain iAs concentrations of 27.2 μg kg^–1^ under AWD and 34.1 μg kg^–1^ under flood) to the low iAs accumulator parent, Lemont (mean grain iAs levels of 34.7 μg kg^–1^ under AWD and 46.4 μg kg^–1^ under flood). Genotypic differences were also observed within the TIL population, with TIL455 and TIL634 displaying a mean difference of 40 μg kg^–1^ under flood and 50 μg kg^–1^ under AWD, respectively.

The 10 genotypes, two parents and eight TILs, grown across all experiments showed consistent patterns (i.e., high accumulators had high grain iAs concentrations, and low accumulators had low grain iAs concentrations) across both field years and greenhouses under flood and AWD conditions (Figure 4). However, the magnitude of grain iAs accumulation differed notably between the field and greenhouse studies. This could be a result of differences in biomass traits between field and greenhouse-grown plants (Barnaby et al., unpublished). We found mature panicle and tiller numbers, proxies for total plant biomass, were both higher in the field compared to the greenhouse (Supplementary Figures 12, 13, respectively).

Root biomass, physiology, and aeration capacity are also known to impact total and iAs accumulation in rice grain (Wu et al., 2011, 2012; Jia et al., 2014). Jia et al. (2014) compared As uptake in two rice cultivars with differing soil oxygenation rates. They found that the genotype with greater oxygenation capacity had lower As uptake overall compared to the genotype with lower oxygenation capacity likely because the higher oxygenation rate allowed for aerobic microbial processes to oxidize As, making it unavailable for rice uptake (Jia et al., 2014). Although we did not measure root biomass in this study, it has been shown that root biomass and oxygenation rates differ by genotype (Wu et al., 2012; Barnaby et al., 2019) and environment (i.e., greenhouse vs. field) (Barnaby et al., unpublished), and it is likely that similar genetic and environmental differences may have impacted grain iAs differences observed between field and greenhouse-grown rice.

Interestingly, we saw a strong, positive correlation between yield and grain iAs concentrations in the greenhouse study under AWD (Figure 6). Norton et al. (2009) found a similar positive correlation between yield and grain total As concentrations for rice grown at two field sites (Norton et al., 2009). However, our field results showed no clear relationship between yield and grain iAs concentrations (Supplementary Figure 12). The controlled greenhouse experiment, where each pot was monitored to precisely meet the AWD20 target, contrasted with field conditions, where heterogeneity across a large field can result in plants experiencing very different soil VWCs under AWD and in turn impacting both yield and grain iAs. Thus, although the greenhouse study is not necessarily indicative of what may happen in the field, it did underscore the relationship between grain yield and iAs concentrations previously identified by Norton et al., which was particularly strong under AWD conditions in this study.

### QTL Impacts on Grain iAs Content

We identified seven QTLs in the TIL mapping population using the grain iAs data from the field 2013 and 2014 flood treatments (Figure 1). One of these QTLs (C9_18034390) was also identified in the 2013 Safe-AWD data, but none were identified from the 2014 AWD30 experiment. However, for the Safe-AWD in 2013, the soil was dried to a target VWC between 35 and 40%, which is only slightly below field saturation; thus, it is not surprising that the QTL identified under this treatment was also found in the flooded field studies. Similar to the QTL identification in our study, Zhang et al. (2014) found more significant QTLs associated with grain total As levels under flooded conditions than under non-flooded conditions (Zhang et al., 2014). This suggests the identified QTLs lack stability across years, which is not uncommon as previous studies have found QTLs can be unstable for several reasons including variations in flowering time or yield by year (Norton et al., 2014, 2019). However, one QTL (C9_18034390) was found in both flood and Safe-AWD treatments, which suggests this QTL is more stable. It has been suggested that even small-effect loci that are environmentally variable may be used to breed low iAs lines through genomic selection as opposed to traditional marker assisted selection (Norton et al., 2019). Thus, identifying putative iAs affecting QTLs regardless of the size of their effect has the potential to inform future breeding efforts.

Wang et al. (2020) found that several QTLs for total As in the grain were associated with those controlling As in the straw and that there was a positive relationship with straw and grain total As contents. Although no straw iAs measurements were made in this study, this may suggest that limiting uptake of As in the vegetative portion of the plant may be one means of decreasing grain accumulation. However, identification of QTLs related to sequestration of As in vegetative tissue may be another means of increasing the effectiveness of breeding efforts (Heuschele et al., 2017).

The TILs selected for the greenhouse study represent genotypes with low to high levels of grain iAs accumulation and contained differing numbers and combinations of QTLs. Although the identified QTLs did not overlap between the 2013 flood and 2014 flood datasets, we were still able to confirm that TILs harboring these QTLs, regardless of the treatment or year they were identified in, resulted in differences in grain iAs levels in the greenhouse study. These results support our finding from the field data that the identified QTLs are indeed affecting grain iAs.

QTLs affecting grain total As accumulation have previously been identified in the TIL mapping population and other cultivars; however, to the authors’ knowledge, no previous study has identified QTLs affecting the more toxic iAs in rice grain (Norton et al., 2014; Zhang et al., 2014; Pinson et al., 2015). The total As QTLs previously identified have some overlap with the iAs QTLs identified in this study. Thus, we further explored these regions for genes that may be responsible for iAs uptake and incorporation. We found five candidate genes within the identified QTL regions (Figure 1). Candidate genes are genes that are suspected of being causal based on their annotated function; however, the QTL regions are large and contain many other potentially causal genes. *OsABCG22* (*LOC_Os09g29660*), an ATP-binding cassette (ABC) transporter gene, was identified in the region of QTL C9_18034390. This gene belongs to the ABCG-WBC cluster, which has been shown to be upregulated by submergence (i.e., flooding) and high concentrations of As (Nguyen et al., 2014). Genes involved in panicle and seed development were colocalized with grain iAs-affecting QTLs on chromosomes 4 and 11, respectively. The *OsPLL4* gene (*LOC_Os04g05050*), a pectate lyase-like gene colocated with QTL C4_2481896, is highly expressed during panicle development (Zheng et al., 2018), whereas *OsOFP18* (*LOC_Os11g05770*), colocated with QTL C11_2659978, is expressed both during seed development and during the young root stage (Yu et al., 2015) and thus could be involved in both the uptake and storage of As. Two genes related to stress tolerance were also found. The germin-like protein (GLP) gene *OsGLP8-1* (*LOC_Os08g08920*) was identified within the QTL region of C8_5186967; GLPs have been shown to colocate with other QTLs associated with drought and blast resistance (Mahmood et al., 2007; Davidson et al., 2010). *OsWRKY64* (*LOC_Os12g02450*) has been found to be upregulated under conditions of iron excess/toxicity and is thought to regulate root elongation as well as shoot growth under stress (Berri et al., 2009; Viana et al., 2017). The colocalization of transporter genes, plant development genes, and stress response genes with the identified iAs-affecting QTLs suggests that grain iAs accumulation is a result of many mechanisms spread across the rice genome.

Across both field and greenhouse experiments, grain iAs accumulation was impacted by the presence or absence of specific QTLs with positive or negative additive effects and specific QTL combinations. The two TILs selected for the greenhouse study that contained no QTLs, TIL596.11 and TIL643, did not differ in grain iAs concentrations from Lemont, which likewise lacked any of the identified iAs-affecting QTLs. This indicates that any additional background TeQing introgressions in the two TILs did not impact grain iAs accumulation. Two QTLs from TeQing introgressions, C11_2659978 and C4_2481896, result in low grain iAs phenotypes, and the other five QTLs result in high iAs phenotypes. Genotypes that contained C9_18034390 had higher mean iAs concentrations as compared to those without (Supplementary Figure 7). Greater than 30% of the highest iAs accumulating group contained C9_18034390 across the field study years and treatments (Figure 2). The QTL combination affecting grain iAs concentrations is most evident when comparing Lemont to TIL455 and TIL552. TIL455 and TIL552 each possess one of the two QTLs that negatively affect grain iAs (C11_2659978 and C4_2481896, respectively), resulting in lower mean grain iAs concentration under both flood and AWD treatments in the greenhouse study as compared to Lemont. The other six TILs possessing QTLs positively affecting iAs levels had mean grain iAs concentrations greater than either TIL455 or TIL552 regardless of irrigation treatment.

We also observed a cumulative effect on grain iAs concentrations regardless of the specific QTL or QTL combination, particularly under flood conditions (Figure 3). This suggests individual QTL effects on iAs grain concentrations may be small compared to the additive effect of containing a combination of several QTLs. Additionally, TILs in the lowest 0–25% of grain iAs concentrations contained fewer of the positive iAs regulating QTLs overall and were more likely to have the negative iAs regulating QTL C11_2659978 and C4_2481896 (Figure 2). In contrast, the percent of genotypes containing a positive iAs regulating QTL in the highest iAs group (75–100%) was greater when compared to the lower iAs groups (<75%). Thus, although we did observe that specific QTLs affect grain iAs accumulation, our results indicate that the cumulative impact of several QTL introgressions associated with an increase in grain iAs can outweigh the impact of any individual QTL.

In contrast to previous studies that have primarily compared varieties that are genetically diverse, our study used a CSSL mapping population generated by backcrossing an F1 hybrid, TeQing, with the recurrent parent, Lemont, leading to targeted TeQing introgressions across the population. The common genetic background of the CSSL population allowed us to identify introgressions on six chromosomes that impact grain iAs concentrations. The fact that QTLs were identified across several chromosomes in this CSSL population indicates that genetic control of this trait is not by one or a few genes but is more widely dispersed throughout the rice genome. Indeed, these results suggest that multiple small-effect QTLs contribute to grain As accumulation, making them difficult to uncover particularly in the case of environmental variability. However, although identifying more iAs affecting QTLs could be beneficial to inform future selective breeding for grain iAs mitigation, we have identified seven QTLs in this CSSL population that can be used as selectable markers to produce new cultivars with low grain iAs content.

Both TIL552 and TIL455 each contained one of the negative iAs regulating QTLs (C11_2659978 and C4_2481896, respectively), resulting in consistently lower grain iAs concentrations when introgressed into Lemont, which has a moderately low iAs grain phenotype. Additionally, neither TIL had significantly reduced yield under AWD20. However, yields for TIL455 were higher under AWD (greenhouse mean of 35 g) compared to TIL 552 (greenhouse mean of 13 g), indicating TIL455 may hold promise for future studies focused on breeding low iAs varieties that are robust to severe AWD treatments.

## Conclusion

Grain iAs accumulation is dependent on both irrigation management practices and genotypic differences. While a severe AWD will reliably reduce grain iAs accumulation, incorporation of selected QTLs can ensure genetic mitigation of iAs, which is particularly important under field production conditions where soil moisture conditions may vary because of management systems or weather conditions. Ultimately, coupling AWD, or other water-saving irrigation management practices, with the deployment of low iAs accumulating cultivars is key to reducing rice iAs exposure while maintaining crop productivity.

## Data Availability Statement

The original contributions presented in the study are included in the article/Supplementary Material, further inquiries can be directed to the corresponding author/s.

## Author Contributions

CF-B, JB, and AM conceived and designed the experiments. CF-B and JB performed the experiments. CF-B, JB, JE, and AM analyzed the data. JB, AM, EC, and VR provided the financial and technical assistance. CF-B wrote the initial manuscript. CF-B, AM, JE, EC, VR, and JB approved the manuscript for publication. All authors contributed to the article and approved the submitted version.

## Conflict of Interest

The authors declare that the research was conducted in the absence of any commercial or financial relationships that could be construed as a potential conflict of interest.

## Footnotes

1 www.ars.usda.gov/GSOR

2 https://www.ars.usda.gov/southeast-area/stuttgart-ar/dale-bumpers-national-rice-research-center/docs/weather-stations/

3 http://www.isbreeding.net/

4 http://www.isbreeding.net
