# Supplementary material for: Grain Inorganic Arsenic Content in Rice Managed Through Targeted Introgressions and Irrigation Management
Source: Front Plant Sci. 2021 Jan 25;11:612054. doi: 10.3389/fpls.2020.612054 (PMC7868431; doi:10.3389/fpls.2020.612054)
Supplement: Supplementary file 1 [file Data_Sheet_1.docx]

Supplementary Material

# Supplementary Data

## Supplementary Figures


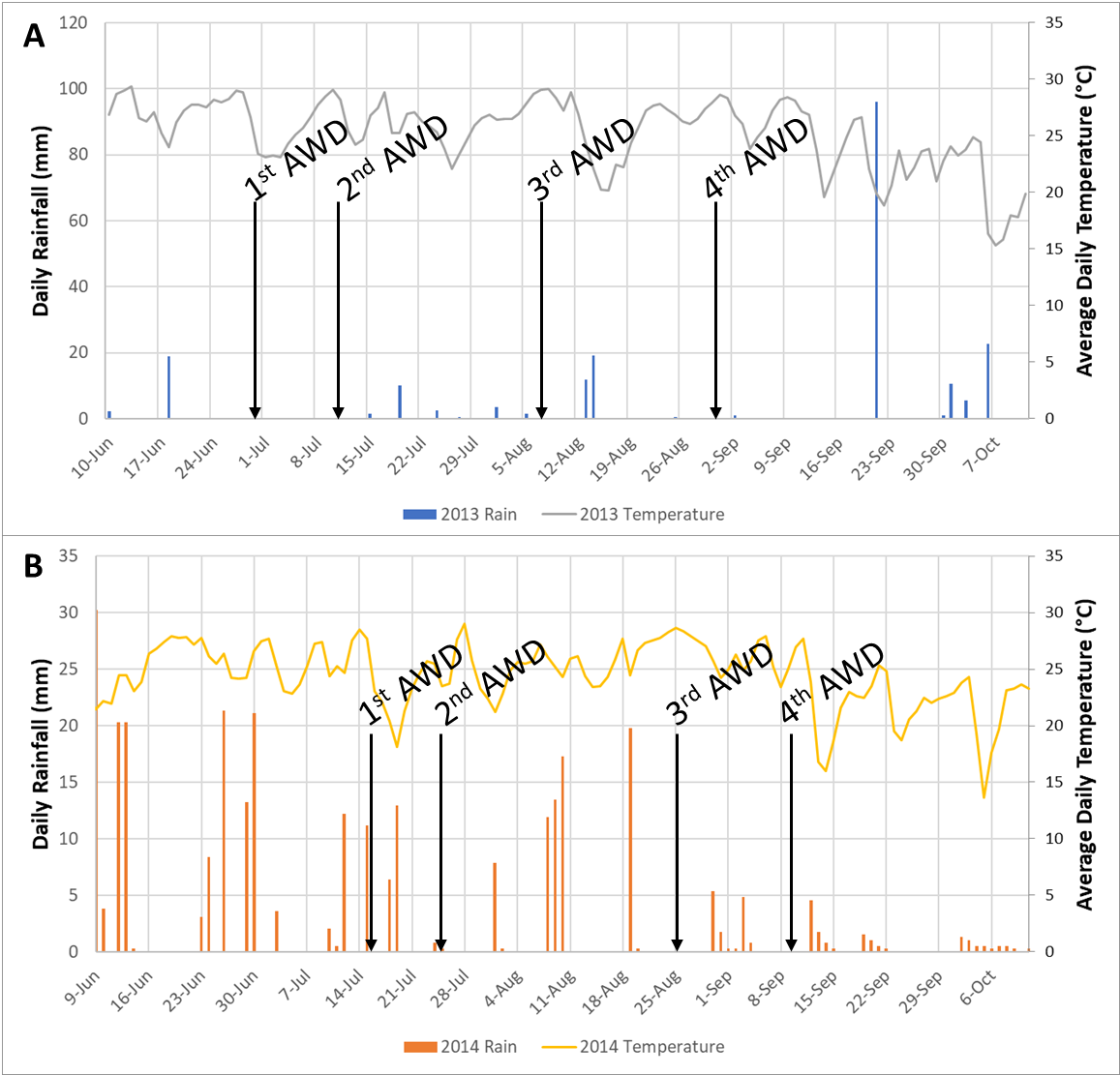


**Supplemental Figure 1**. Field conditions for 2013 (A) and 2014 (B) including approximate dates for the start of each alternate wetting and drying (AWD) period.

**
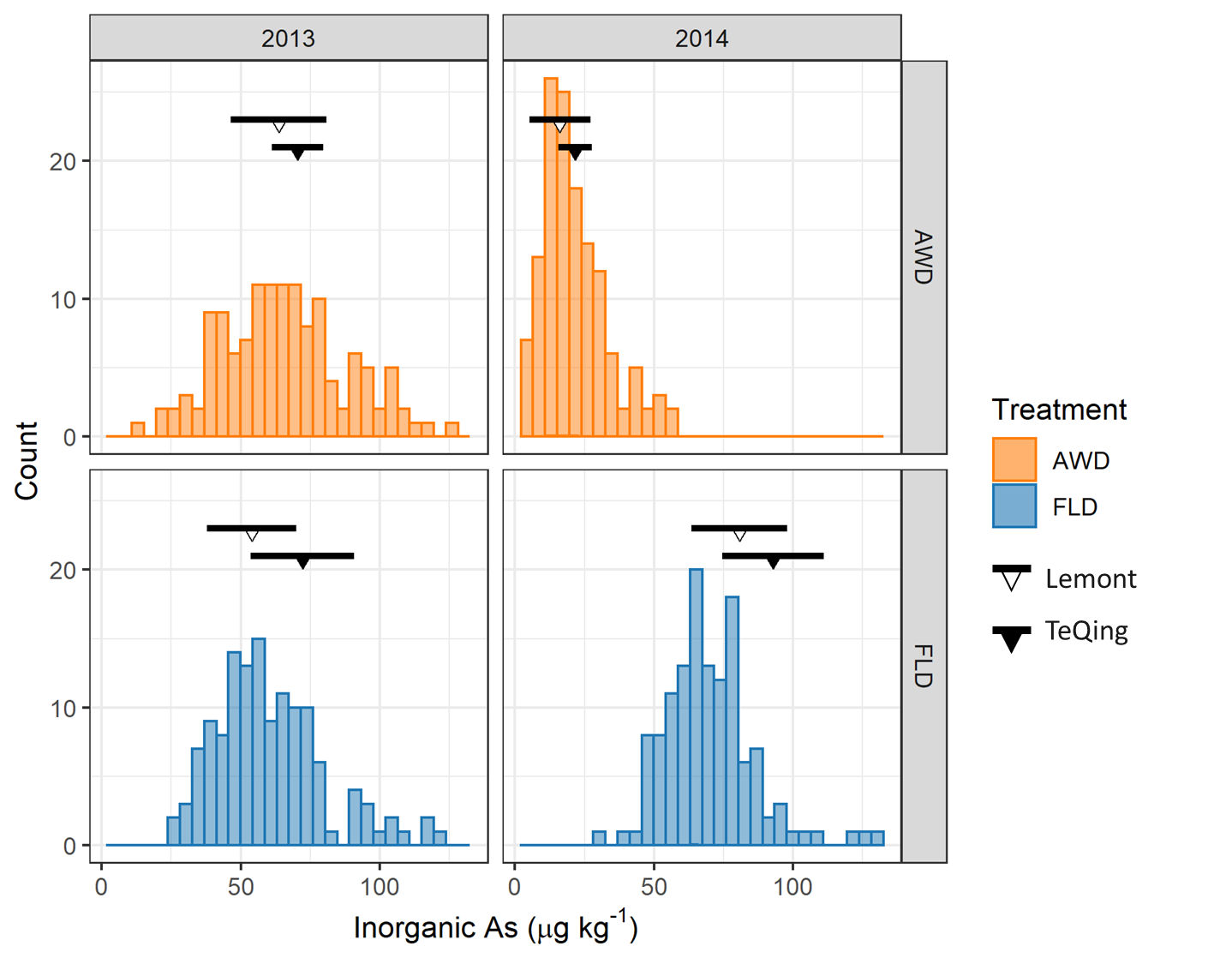
**

**Supplemental Figure 2.** Frequency distribution of grain inorganic concentrations in brown rice among TIL population in 2013 and 2014 under AWD and Flood (FLD) treatments. Means (± 1 standard deviation) for the parents, Lemont (open triangle) and TeQing (black triangle) are shown for the six biological replicates. Average target soil volumetric water content (VWC) was 35-40 % (Safe AWD) in 2013 and 25-30% (AWD30) in 2014.


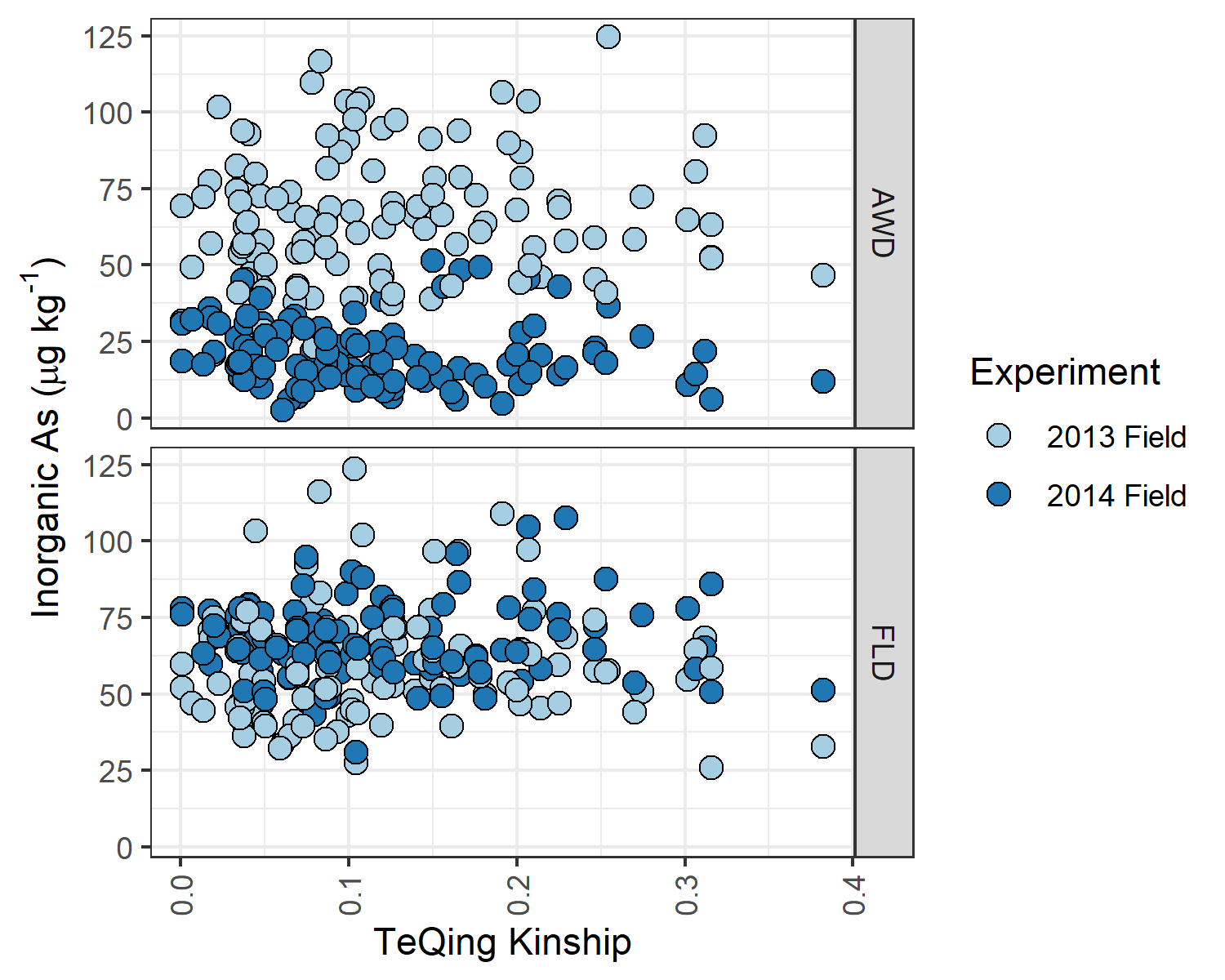


**Supplemental Figure 3.** TeQing kinship, expressed as the fraction of a TIL’s genome from TeQing, within TIL mapping population vs. measured inorganic As in brown rice for both field study years.


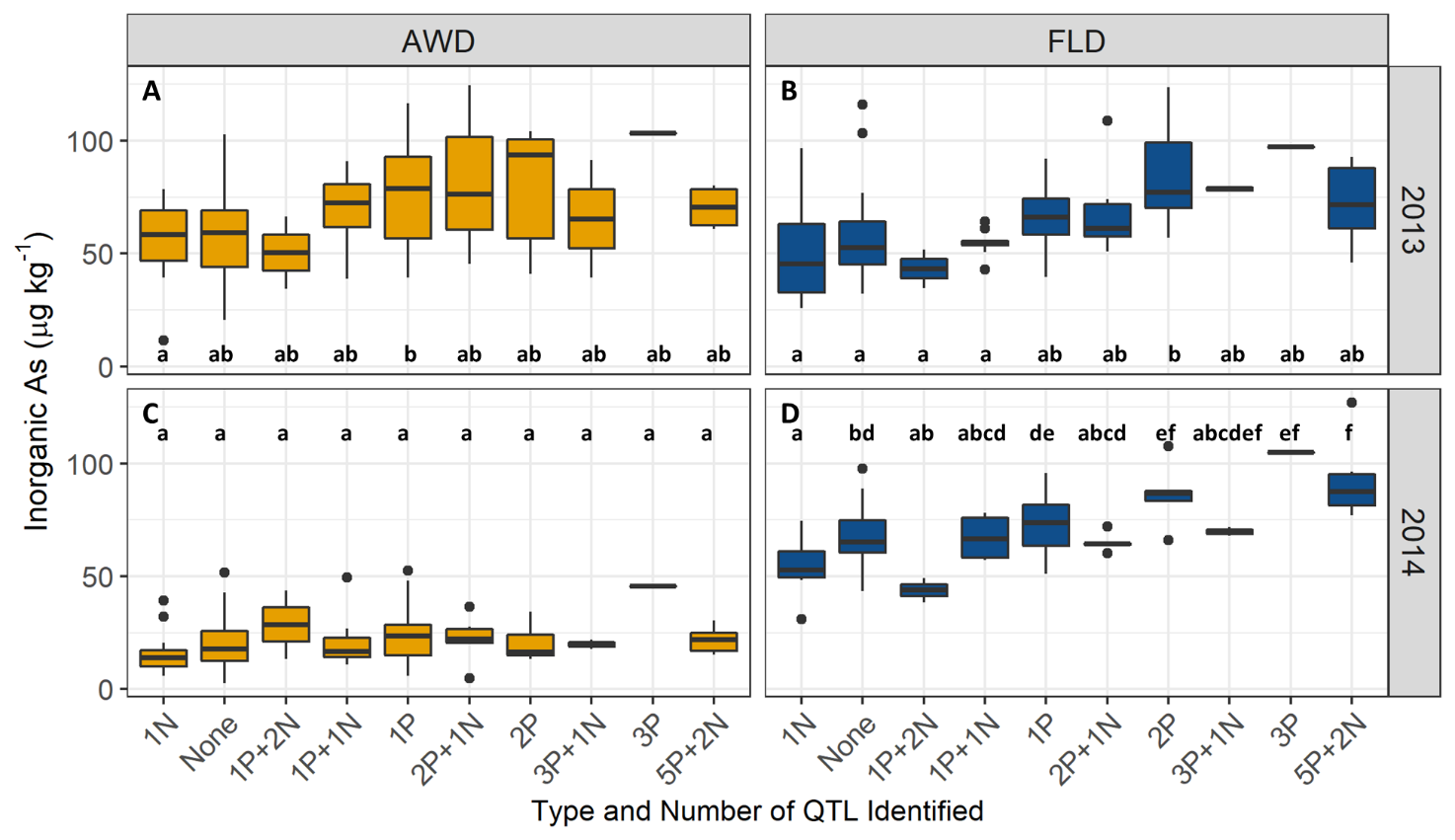


**Supplemental Figure 4.** Brown rice inorganic As concentrations (µg kg^-1^) in field 2013 under alternate wetting and drying (AWD) (A) and flooded (FLD) (B) and 2014 AWD (C) and flood (D) irrigation management treatments by number of quantitative trait loci (QTL) across entire CSSL mapping population. Safe-AWD was used in 2013 and AWD30 was used in 2014. Only one biological replication of the genotype containing 3 positive iAs affecting QTL was grown in 2013 and 2014, all other QTL groups have a minimum of 2 biological replicates. The parents Lemont and TeQing are included in the categories “none” and “5P+2N” respectively. Letters indicate significant differences within each treatment and year (*p* < 0.05).


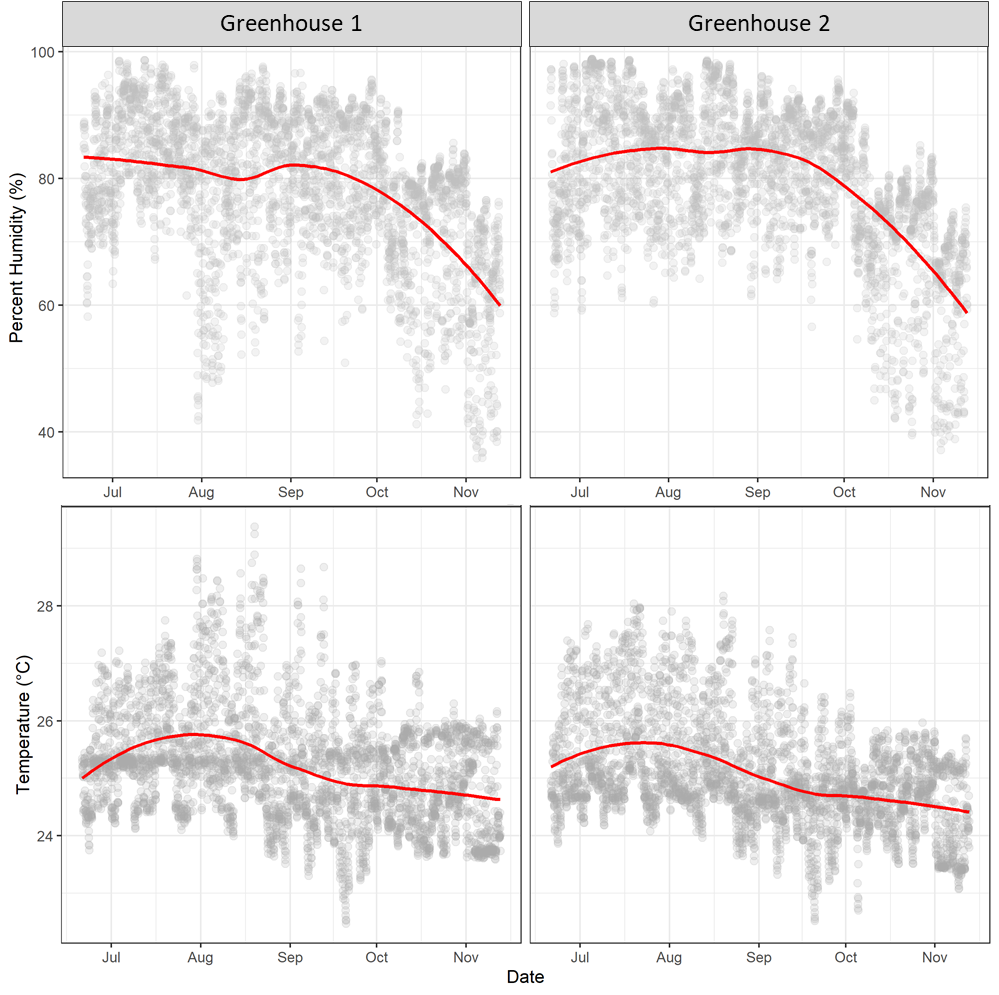


**Supplemental Figure 5.** Percent humidity and air temperature in each greenhouse over the course of the experiment.


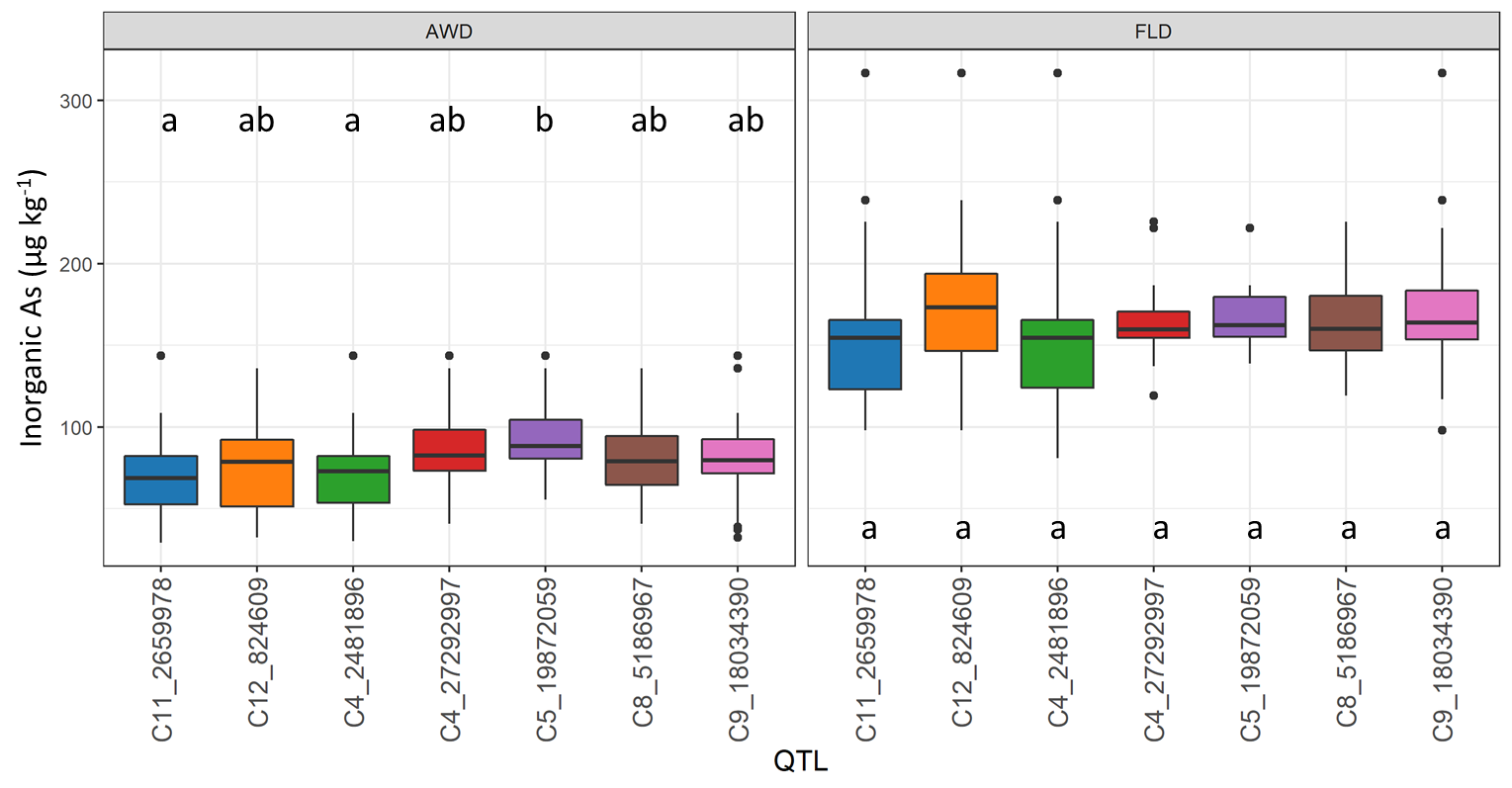


**Supplemental Figure 6.** Brown rice inorganic As concentrations (µg kg^-1^) within the 2019 greenhouse study by QTL for flood (FLD) and alternate wetting and drying (AWD) irrigation methods. Each QTL represents a minimum of 8 biological replicates. Statistically significant differences between group means for inorganic As were determined by one-way ANOVA *(p <* 0.05). Letters indicate statistically significant differences by Tukey HSD (*p* < 0.05).


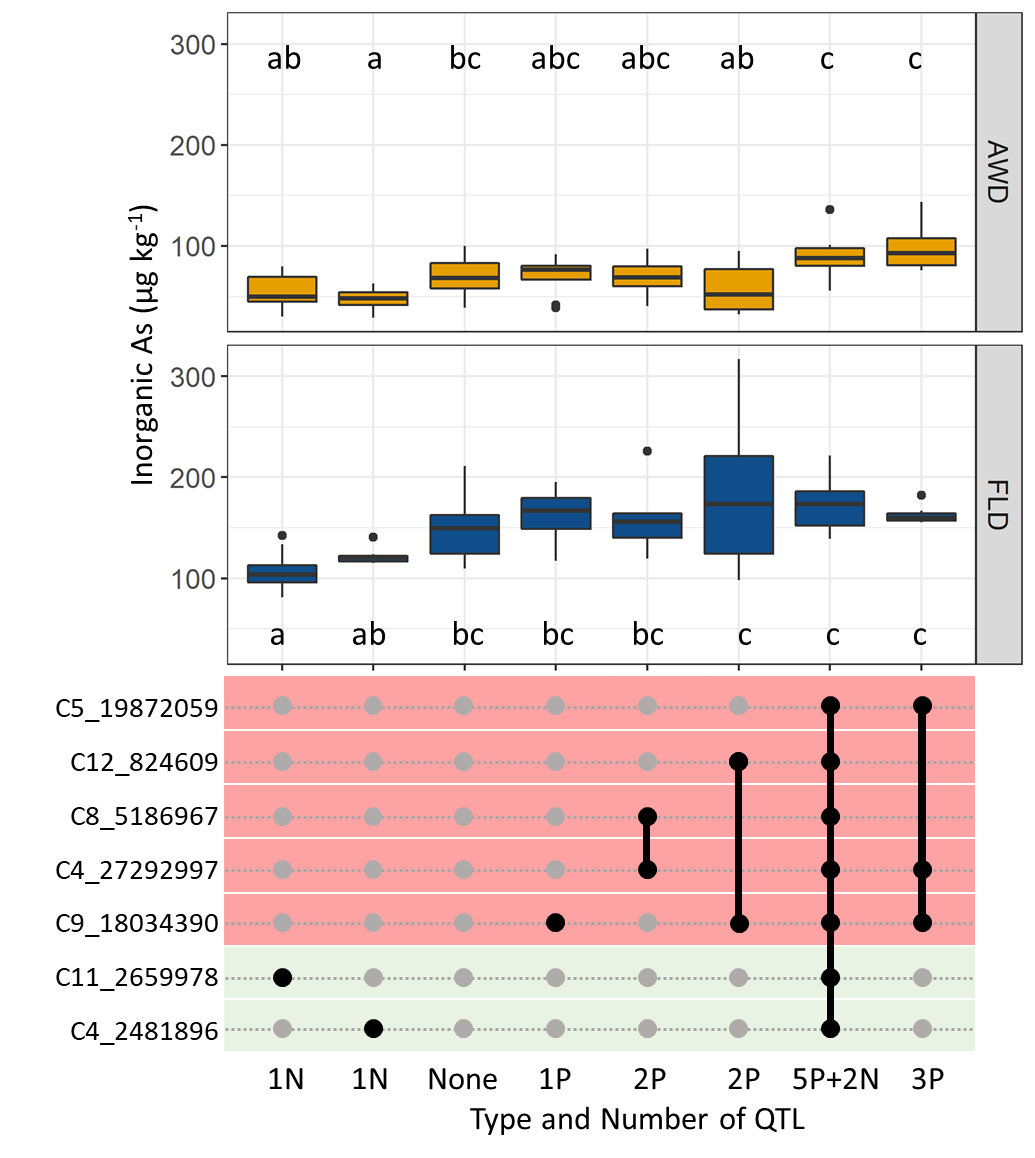


**Supplemental Figure 7.** Brown rice inorganic As concentrations (µg kg^-1^) under flooded (FLD) and alternate wetting and drying (AWD) irrigation management treatments by type and number of quantitative trait loci (QTL) combinations found in the 8 TILs and 2 parents, Lemont and TeQing, selected for the 2019 greenhouse experiment. QTL have either positive (P, red color) or negative (N, green color) effect on grain iAs levels, “None” indicates no introgressions at these QTL locations. Each QTL group represents a minimum of 8 biological replicates. Letters indicate statistically significant differences by Tukey HSD (*p* < 0.05).


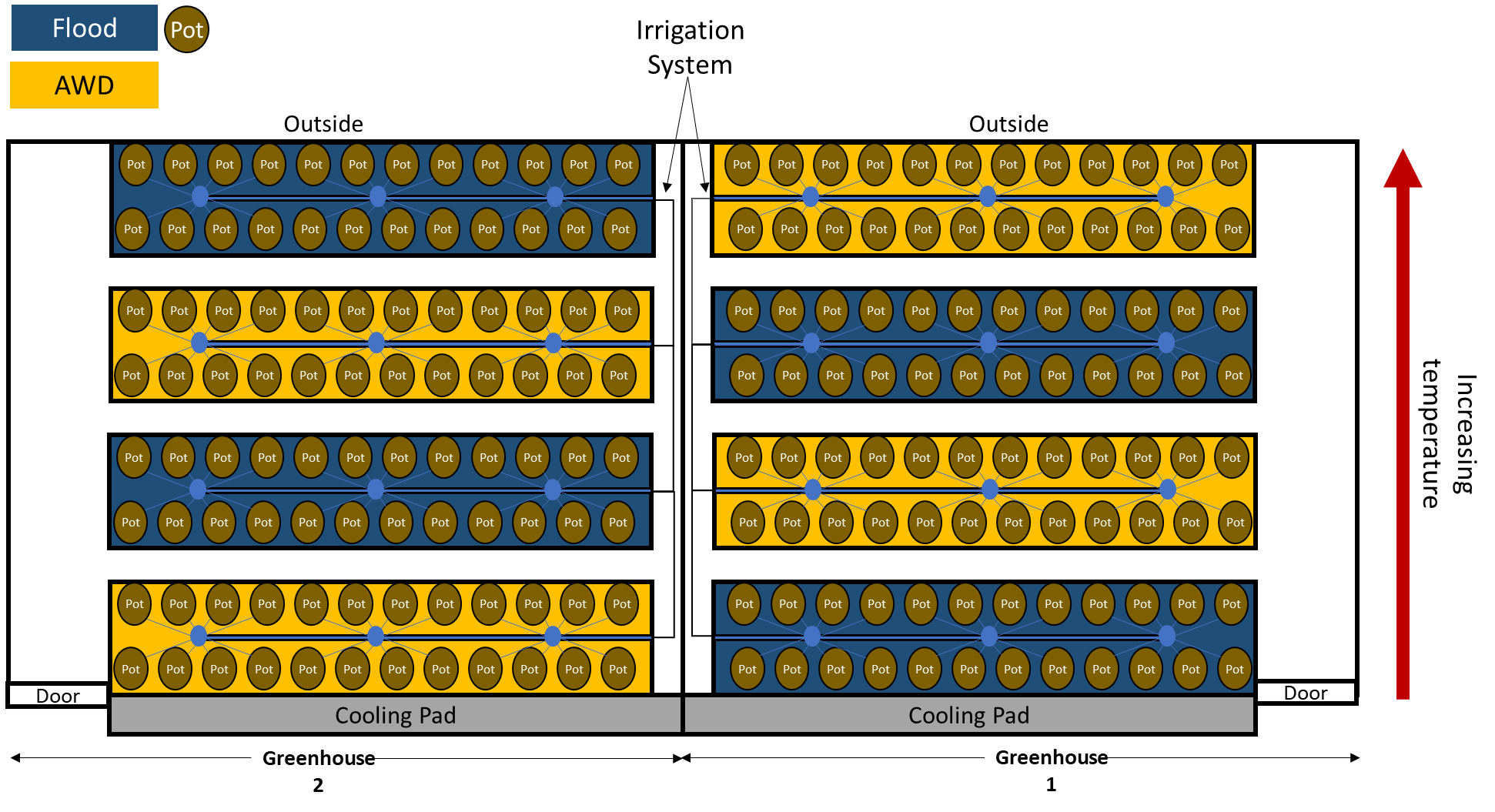


**Supplemental Figure 8.** Greenhouse study setup, showing distance from the cooling pad of AWD and flood treatments in greenhouse 1 and 2 and the resulting temperature gradient.


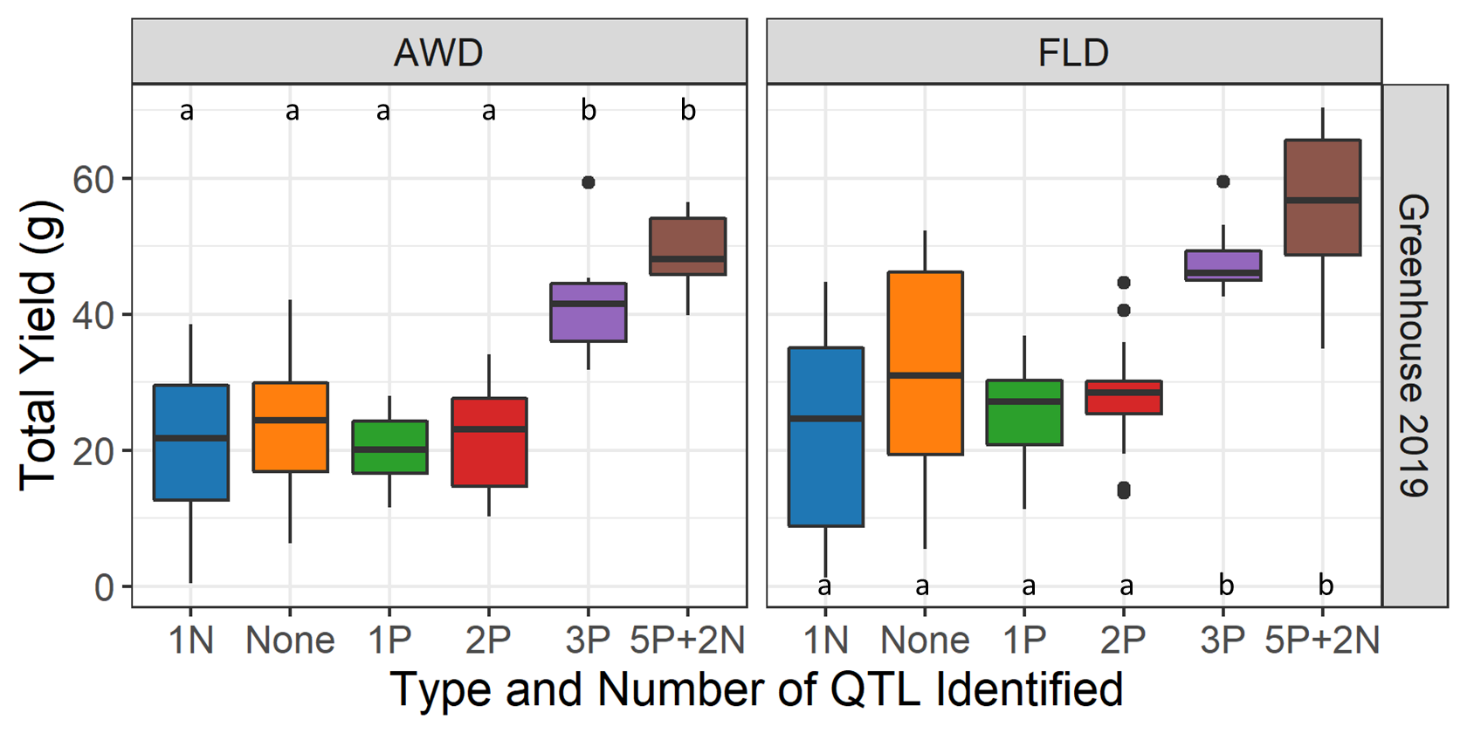


**Supplemental Figure 9.** Total grain yields for 2019 greenhouse experiment by QTL number and type under flood (FLD) and alternate wetting and drying (AWD) irrigation treatments. Each QTL group is represented by one or more genotypes, each with 8 biological replicates. Statistically significant differences between group means for total yield were determined by one-way ANOVA *(p <* 0.05). Letters indicate statistically significant differences by Tukey HSD (*p* < 0.05).


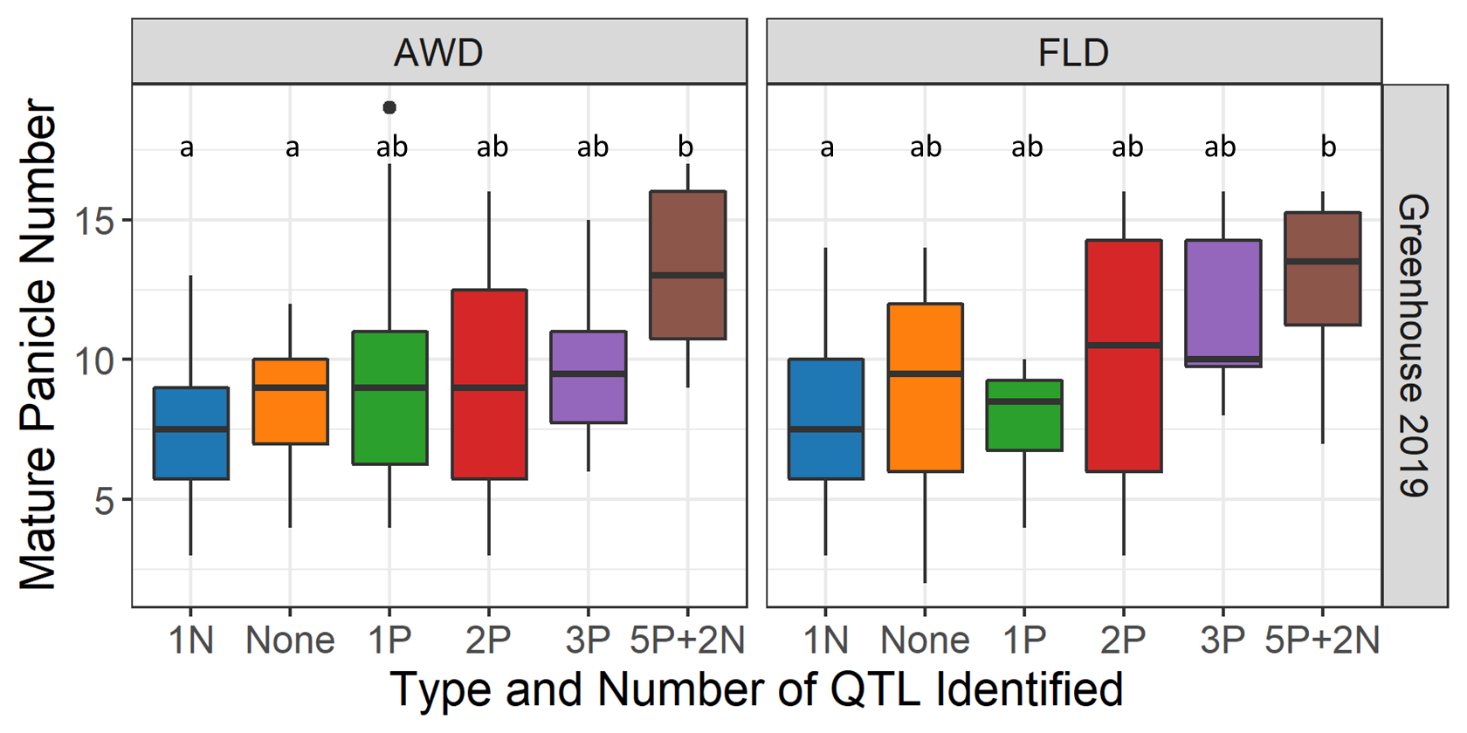


**Supplemental Figure 10.** Total mature panicle number for greenhouse 2019 experiment by QTL number and type under flood (FLD) and alternate wetting and drying (AWD) irrigation treatments. Each QTL group is represented by one or more genotypes, each with 8 biological replicates. Statistically significant differences between group means for number of mature panicles were determined by one-way ANOVA *(p <* 0.05). Letters indicate statistically significant differences by Tukey HSD (*p* < 0.05).


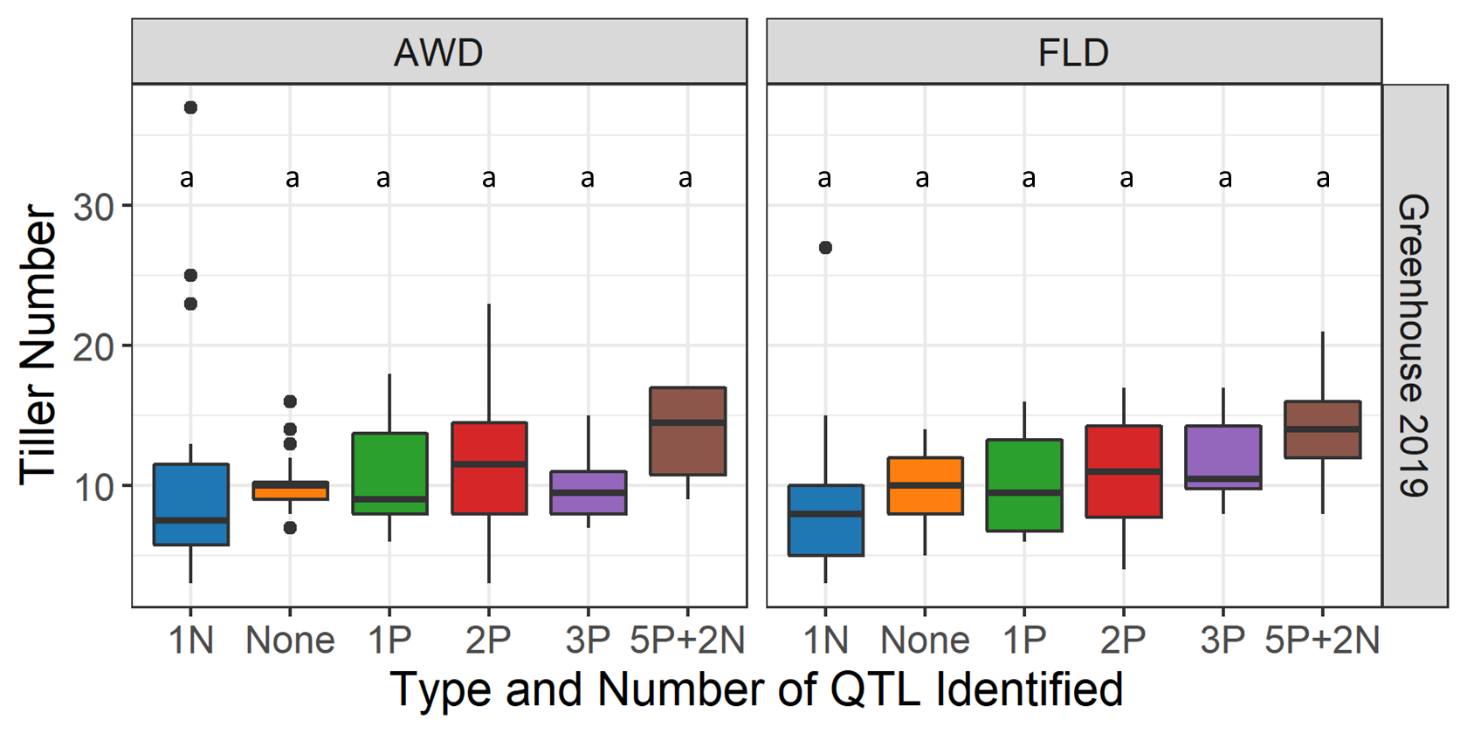


**Supplemental Figure 11.** Total tiller number for greenhouse 2019 experiment by QTL number and type under flood (FLD) and alternate wetting and drying (AWD) irrigation treatments. Each QTL group is represented by one or more genotypes, each with 8 biological replicates. Statistically significant differences between group means for number of tillers were determined by one-way ANOVA *(p <* 0.05). Letters indicate statistically significant differences by Tukey HSD (*p* < 0.05).


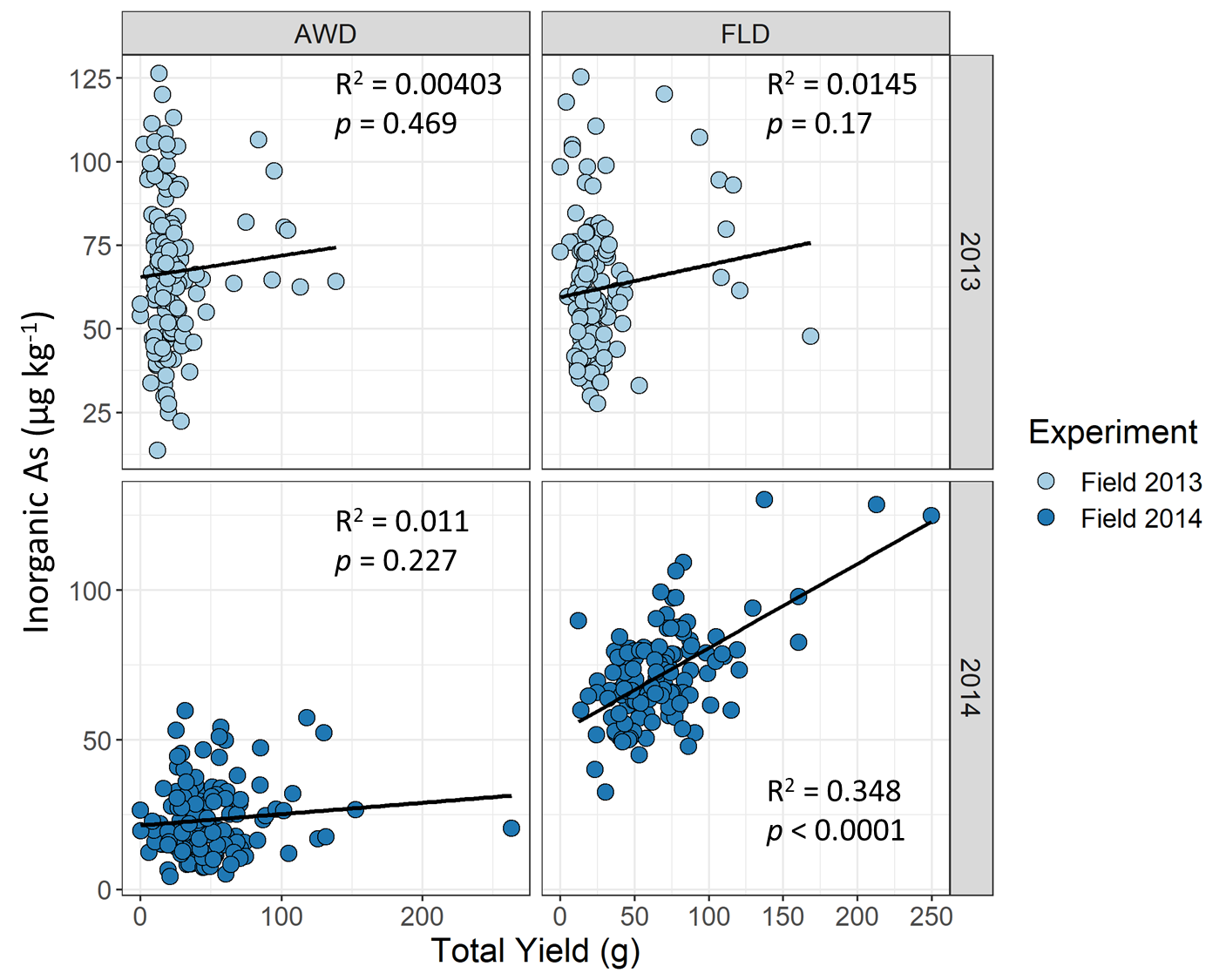


**Supplemental Figure 12.** Field 2013 and 2014 grain inorganic As concentrations by total grain yield for flood (FLD) and alternate wetting and drying (AWD) irrigation methods.


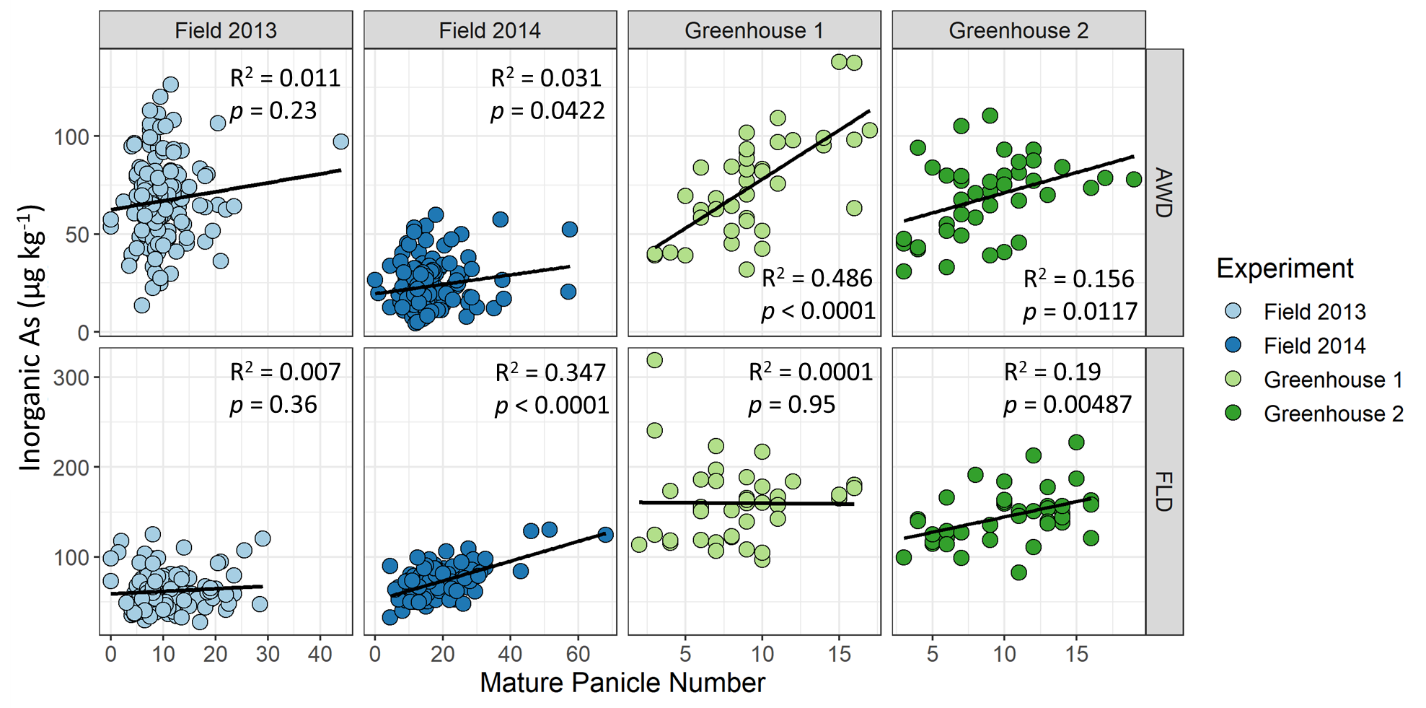


**Supplemental Figure 13.** Field 2013 and 2014 and greenhouse 2019 grain inorganic As concentrations by number of mature panicles at harvest for flood (FLD) and alternate wetting and drying (AWD) irrigation methods.


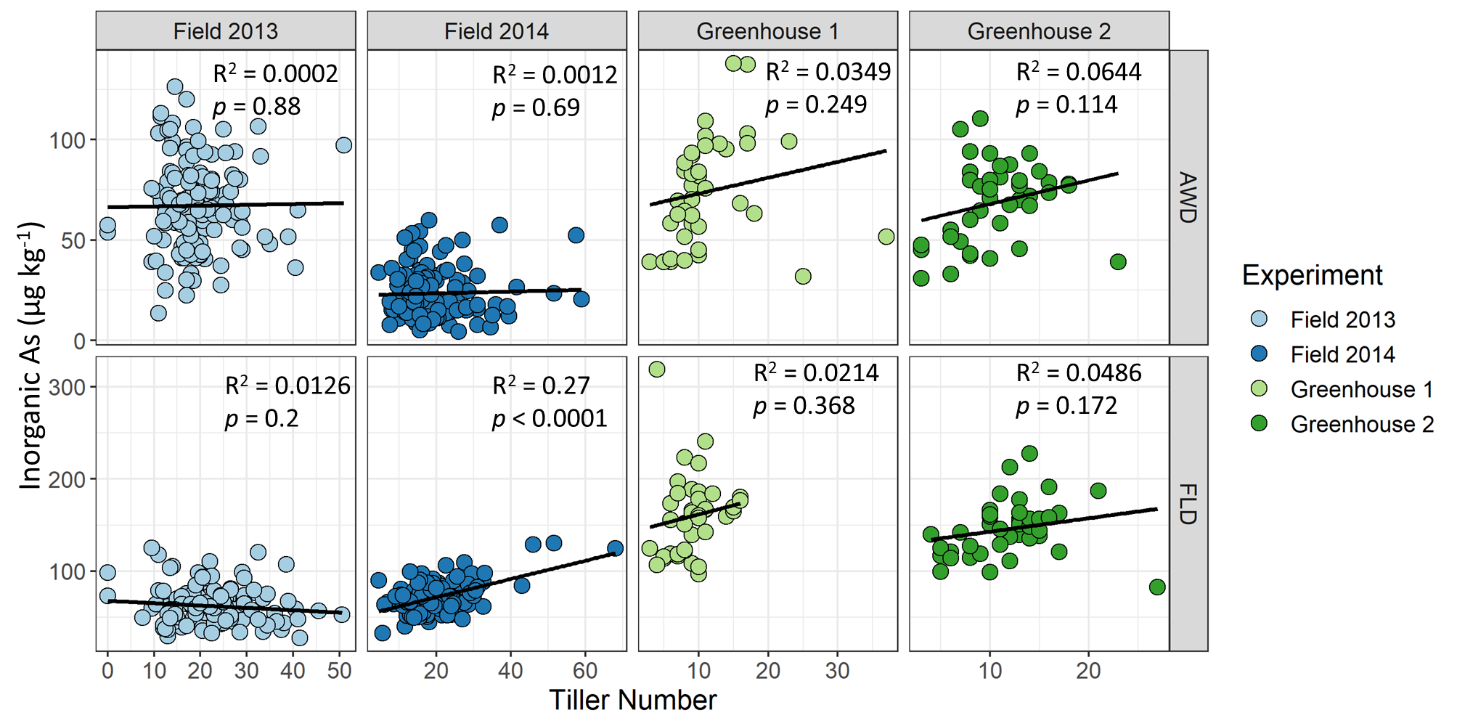


**Supplemental Figure 14.** Field 2013 and 2014 and greenhouse 2019 grain inorganic As concentrations by tiller number at harvest for flood (FLD) and alternate wetting and drying (AWD) irrigation methods.


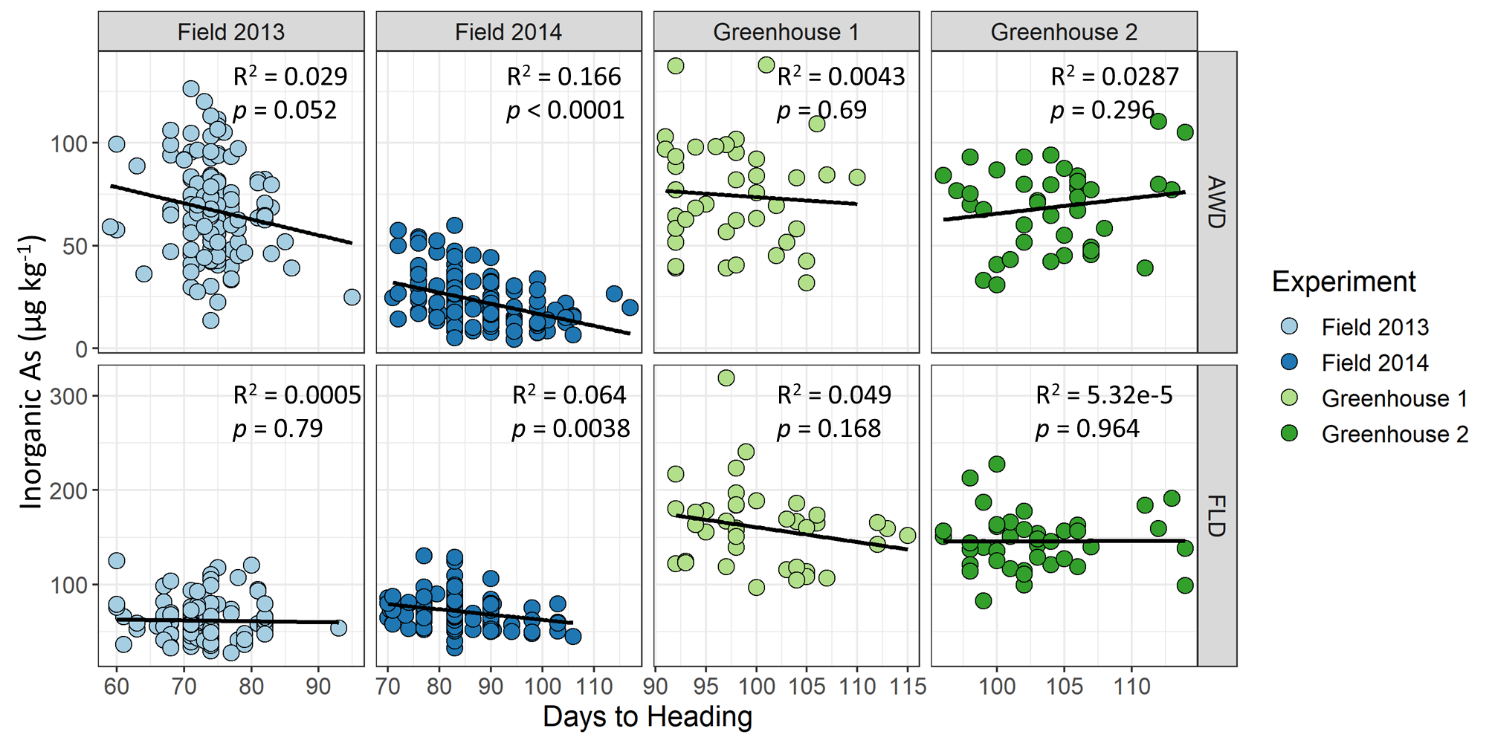


**Supplemental Figure 15.** Field 2013 and 2014 and Greenhouse 2019 grain inorganic As concentrations by days to heading for flood (FLD) and alternate wetting and drying (AWD) irrigation methods.

## Supplementary Tables

**Supplemental Table 1.** Identified quantitative trait loci (QTL) affecting brown rice grain inorganic As concentrations. “Dataset Used” column indicates the brown rice inorganic As data used (treatment and year). Marker name indicates the chromosome and position for each QTL (e.g. chromosome 8 position 5186967 is C8_5186967). PVE (%) is percentage phenotypic variation explained and LOD is logarithm of the odds ratio.

| **Dataset Used** | **QTL Name** | **LOD** | **PVE (%)** | **Additive Effect** |
| --- | --- | --- | --- | --- |
| Flood_2013 | C8_5186967 | 3.5332 | 10.7153 | 10.1307 |
| Flood_2013,  Safe AWD 2013 | C9_18034390 | 3.8825 | 11.876 | 9.7187 |
| Flood_2014 | C4_2481896 | 3.863 | 6.0793 | -6.1127 |
| Flood_2014 | C4_27292997 | 8.0766 | 13.8986 | 8.1675 |
| Flood_2014 | C5_19872059 | 3.6108 | 5.6527 | 5.6912 |
| Flood_2014 | C11_2659978 | 8.0706 | 13.9859 | -7.6105 |
| Flood_2014 | C12_824609 | 4.9947 | 8.0494 | 8.4488 |

**Supplemental Table 2.** Percent change in total yield under flood and alternate wetting and drying (AWD) irrigation treatments observed in field and greenhouse studies. The percent change was calculated from mean grain yields under flood and AWD for 4 biological replicates in each experiment. Asterisk (*) shows significant decreases in yield under AWD based upon the mean comparison.

|  |  |  |  | Percent Change in Mean Total Grain Yield (g) between AWD to Flood Treatments | | | | | | | |
| --- | --- | --- | --- | --- | --- | --- | --- | --- | --- | --- | --- |
| Type | Name | iAs Phenotype |  | Field 2013 |  | Field 2014 |  | Greenhouse 1 |  | Greenhouse 2 |  |
| Parent | Lemont | Parent |  | -15.7 |  | -24.8 |  | -26.5 |  | -24.3 |  |
| TIL | 455 | Low |  | +42.4 |  | -30.9 |  | -13.8 |  | -27.2 |  |
| TIL | 552 | Low |  | -48.2 |  | -11.9 |  | +93.3 |  | +10.0 |  |
| TIL | 596.11 | Low |  | +25.1 |  | -30.8 |  | +55.2 |  | -29.1 |  |
| TIL | 643 | Low |  | -20.7 |  | -29.5 |  | -24.8 |  | -27.6 |  |
| TIL | 389 | High |  | -28.3 |  | -52.7 |  | -5.7 |  | -30.1 |  |
| TIL | 604.11 | High |  | +26.6 |  | -8.4 |  | -26.6 |  | -13.4 |  |
| TIL | 381.11 | High |  | -25.6 |  | -12.7 |  | -17.6 |  | **-29.8*** |  |
| TIL | 634 | High |  | +18.7 |  | -10.8 |  | -8.7 |  | -18.6 |  |
| Parent | TeQing | Parent |  | +2.8 |  | -10.4 |  | -5.5 |  | -19.8 |  |

**p* < 0.05
